# Identification of microRNAs Implicated in Modulating Senecionine-Induced Liver Toxicity in HepaRG Cells

**DOI:** 10.3390/foods11040532

**Published:** 2022-02-12

**Authors:** Anne-Margarethe Enge, Heike Sprenger, Albert Braeuning, Stefanie Hessel-Pras

**Affiliations:** Department of Food Safety, German Federal Institute for Risk Assessment, Max-Dohrn-Str. 8-10, 10589 Berlin, Germany; anne-margarethe.enge@bfr.bund.de (A.-M.E.); heike.sprenger@bfr.bund.de (H.S.); albert.braeuning@bfr.bund.de (A.B.)

**Keywords:** cell cycle, IPA, microRNA, *PAK1*, pyrrolizidine alkaloids, senecionine

## Abstract

1,2-unsaturated Pyrrolizidine Alkaloids (PAs) are secondary plant metabolites that occur as food contaminants. Upon consumption, they can cause severe liver damage. PAs have been shown to induce apoptosis, to have cytotoxic and genotoxic effects, and to impair bile acid homeostasis in the human hepatoma cell line HepaRG. The major mode of action of PAs is DNA- and protein-adduct formation. Beyond that, nuclear receptor activation has only been observed for one receptor and two PAs, yielding the possibility that other cellular mediators are involved in PA-mediated toxicity. Here, the mode of action of Senecionine (Sc), a prominent and ubiquitous representative of hepatotoxic PAs, was investigated by analyzing 7 hepatic microRNAs (miRNAs) in HepaRG cells. Ultimately, 11 target genes that were predicted with Ingenuity Pathway Analysis software (IPA) were found to be significantly downregulated, while their assigned miRNAs showed significant upregulation of gene expression. According to IPA, these targets are positively correlated with apoptosis and cellular death and are involved in diseases such as hepatocellular carcinoma. Subsequent antagomiR-inhibition analysis revealed a significant correlation between PA-induced *miRNA-4434* induction and P21-Activated Kinase-1 (*PAK1*) downregulation. *PAK1* downregulation is usually associated with cell cycle arrest, suggesting a new function of Sc-mediated toxicity in human liver cells.

## 1. Introduction

Pyrrolizidine alkaloids are the most common natural toxins with over 660 different chemical structures. They are present in more than 6000 plant species worldwide and can occur as contaminants in food and feed, posing a possible health risk for humans and livestock [1]. For example, they can be found in products such as tea, honey, culinary herbs, dietary supplements or herbal preparations such as traditional medicine [2,3,4,5]. It must be stressed that the exposure to pyrrolizidine alkaloids can occur via a wide variety of foodstuff, which increases the likelihood of ingestion. Because 1,2-unsaturated Pyrro-lizidine Alkaloids (PAs) have genotoxic properties and are considered to be carcinogenic, no safe threshold value such as the TDI (Tolerable Daily Intake) can be derived. Therefore, it is recommended to apply the ALARA principle (As Low As Reasonably Achievable) to prevent unnecessary exposure [6].

PAs are known to require metabolic activation in the liver, usually provided by members of the cytochrome P450 superfamily [7]. The hepatotoxic metabolites may cause acute toxic effects such as Hepatic Sinusoidal Obstruction Syndrome (HSOS), resulting in ascites, hepatomegaly, and ultimately death. Chronic intoxication may lead to megalocytosis, liver cirrhosis and cancer [7,8,9,10,11,12,13,14,15,16]. To a lesser extent, PAs are also pneumotoxic, most likely due to systemic distribution of reactive metabolites to the lung [17].

Pyrrolizidine alkaloids consist of one so-called necine base, which is esterified with one or two aliphatic mono- or dicarboxylic acids (necic acids). Accordingly, they are classified into different structure types, depending on the structure of their necine base (retronecine-, heliotridine-, otonecine-, and platynecine-type) and further into monoesters, open-chained diesters and cyclic diesters [18].

Until today, many in vitro studies have been conducted showing that PAs cause apoptosis [19,20], cytotoxicity [21], and genotoxicity [22] in a structure-dependent manner. Moreover, they were shown to disturb bile acid homeostasis in the human hepatoma cell line HepaRG [23,24]. Upon PA treatment, it is consistently observed that the expression pattern of genes relevant for apoptosis, bile acid homeostasis, and metabolism is highly deregulated in liver cells in vivo and in vitro [21,23,25]. Nonetheless, some ambiguities remain regarding the underlying molecular mechanisms of PA-mediated hepatotoxicity. The major molecular mode of action is the binding of PA metabolites to DNA and proteins to form adducts via alkylation [7]. Nevertheless, interactions between PAs and specific regulatory elements may pose additional molecular mechanisms leading to deregulation of cellular signaling and disturbance of metabolic pathways. Therefore, PA-mediated nuclear receptor activation was investigated, mainly because nuclear receptors are typical targets in xenobiotic-induced toxicity. However, no PA-induced activation could be observed, with the exception of Pregnane X Receptor (PXR), which was exclusively activated by the PAs echimidine and Lasiocarpine (Lc), resulting in the activation of its target CYtochrome P450 monooxygenase (CYP) 3A4 [26]. Therefore, it appears likely that cellular signaling pathways and mediators other than nuclear receptor interference are involved in mediating the effects of PAs. miRNAs are small, non-coding, 21- to 25-nucleotide-long RNA molecules that regulate a wide variety of physiological processes such as cell growth, development, apoptosis, differentiation, and carcinogenesis at the post-transcriptional level [27,28]. In animals, miRNAs bind their target mRNAs in the 3′-UnTranslated Region (UTR). Depending on the complementarity of base pairing between the miRNA and the mRNA target, the binding of the miRNA either leads to mRNA cleavage or translational repression. It is postulated that translational repression is the predominant mechanism by which metazoan miRNAs negatively regulate their targets [29]. Many miRNAs are crucial regulators of bile acid homeostasis, lipid and glucose metabolism, inflammation, apoptosis, and proliferation [30,31,32], for example, and are deregulated in many liver diseases [32,33]. Notably, CYP7A1, the rate-limiting enzyme in bile acid synthesis, is regulated by two miRNAs, with one of them being the most abundant miRNA in the liver [34]. Interestingly, a correlation between chronic exposure to the PA riddelliine and an altered miRNA expression pattern in the liver has been observed in a 12-week feeding study with rats [35]. Moreover, an integrative analysis studying the miRNA-mRNA interaction after acute incubation with the PA Monocrotaline (Mc) in high doses found the phagosome signaling pathway to be a relevant molecular mechanism of PA-induced HSOS in mice [36]. Some miRNAs are solely expressed in a tissue-specific manner; others circulate in body fluids such as blood or urine and have been proposed as non-invasive biomarkers for disease prediction and progression. For example, circulating miRNAs in blood samples from patients suffering from PA-induced HSOS were positively correlated with the severity of liver injury and progression of HSOS [37]. Additionally, patients suffering from intrahepatic cholestasis showed elevated miRNA levels in blood and liver tissue [33]. Therefore, miRNA signatures are currently discussed as early-stage biomarkers for HSOS and other liver-specific injuries [28,38].

The present study aimed to examine the role of hepatic miRNAs in the regulation of PA-induced hepatotoxicity in HepaRG cells. For this purpose, relevant hepatic miRNAs were discovered that are sensible to PA treatment. With quantitative analysis and Ingenuity Pathway Analysis (IPA), an evidence-based tool for target and pathway prediction, a selection of target genes was further subjected to analysis to identify the potential engaged molecular functions and biological pathways involved in PA-mediated toxicity in human liver cells.

## 2. Materials and Methods

### 2.1. Chemicals

The PAs Lc and senecionine (Sc) (>95% purity) were obtained from PHYTOPLAN Diehm & und Neuberger GmbH (Heidelberg, Germany). In order to get 5 mM stock solutions, the PAs were dissolved in 50% acetonitrile (ACN) and 50% water (*v*/*v*). All other chemicals were purchased from Merck KGaA (Darmstadt, Germany) and Thermo Fisher Scientific (Waltham, MA, USA).

### 2.2. Cell Culture

The human hepatoma cell line HepaRG was purchased from Biopredic International (Saint-Grégoire, France). The cells were cultivated at 37 °C in a humidified atmosphere of 5% CO_2_ [39]. After seeding, cells were cultivated in William’s Medium E with stable glutamine (PAN-Biotech, Aidenbach, Germany) for proliferation. The medium was supplemented with 10% Fetal Bovine Serum (FBS; PAN-Biotech, Aidenbach, Germany), 50 µM hydrocortisone hemisuccinate (Merck KGaA, Darmstadt, Germany), 100 U/mL penicillin and 100 µg/mL streptomycin (Capricorn Scientific, Ebsdorfergrund, Germany) and 5 µg/mL human insulin (PAN-Biotech, Aidenbach, Germany). After two weeks of proliferation, 1.7% of DiMethyl SulfOxide (DMSO; Merck KGaA, Darmstadt, Germany) was added to initiate differentiation and HepaRG cells were cultivated for another two weeks to fully differentiate before being used for experiments.

### 2.3. Liver-Specific miRNA Array

For liver-specific miRNA expression screening, HepaRG cells were seeded at a density of 0.2 × 10^6^ cells per well in 6-well plates and cultivated as described in Section 2.2. After four weeks of cultivation and 48 h prior to incubation, fully differentiated cells were adapted to treatment medium containing 1.7% DMSO and a reduced FBS concentration of 2%. The cells were incubated with 10, 35 and 250 µM of the most potent Lc for 8 h or with 2.5, 10 and 35 µM Lc for 24 h, respectively. The miRNA was isolated using the miRNeasy Mini Kit (Qiagen, Hilden, Germany) according to the manufacturer’s protocol, including a purification step of the miRNA-enriched fraction using the RNeasy MinElute Cleanup Kit (Qiagen, Hilden, Germany). Initially, cells were lysed with 700 µL QIAzol lysis reagent. As a miRNA isolation efficiency control, a spike-in control consisting of three synthetic templates in different concentrations (Qiagen, Hilden, Germany) was added to the lysis buffer. The subsequent procedure was as follows: incubation for 5 min at Room Temperature (RT), addition of 140 µL chloroform per sample and thoroughly mixing, incubation for 3 min at RT, centrifugation for 15 min at 12,000× *g* at 4 °C, transfer of the upper aqueous phase intro a new tube, addition of one volume of 70% ethanol (usually 350 µL) and thorough mixing, transfer of the sample into an RNeasy Mini spin column, and centrifugation for 15 s at 8000× *g* at RT. The RNeasy Mini spin columns containing the mRNA fractions were discarded. The flow-through containing the smaller miRNA fraction was purified as follows: addition of 450 µL 100% ethanol, thorough mixing, transfer of the sample into an RNeasy MinElute spin column and centrifugation for 15 s at 8000× *g* at RT, washing of column with 700 µL RWT buffer and 500 µL RPE buffer, respectively, for 15 s at 8000× *g* at RT, final washing with 500 µL 80% ethanol with subsequent centrifugation for 2 min at 8000× *g* at RT and subsequent drying by centrifugation for 5 min at 8000× *g* at RT. Afterwards, the miRNA fractions were eluted in 14 µL water and quantified at 260 and 280 nm on a TecanM200Pro spectrometer (Tecan Group Lt., Männedorf, Switzerland). Next, 10 ng miRNA (naturally occurring as non-polyadenylated) were polyadenylated by a poly(A) polymerase and reverse transcribed into cDNA using the miRCURY LNA RT Kit (Qiagen, Hilden, Germany) according to the manufacturer’s instructions. Additionally, as a reverse transcription efficiency control, a spike-in control consisting of a synthetic RNA target (Qiagen, Hilden, Germany) was added to the reaction buffer. The reverse transcription step lasted for 60 min at 42 °C, followed by an inactivation step for 5 min at 95 °C. Quantitative real-time Polymerase Chain Reaction (qPCR) on pre-designed PCR panels with Locked Nucleic Acids (LNA)-enhanced oligonucleotides (miRCURY LNA miRNA custom PCR array of 84 liver-specific miRNAs (see Supplementary Materials Table S1 for a list of all miRNAs investigated)) was performed from 62.5 pg cDNA on an ABI 7900HT Fast Real-Time PCR system (Applied Biosystems, Foster City, CA, USA) in 384-well format using miRCURY LNA SYBR Green Master Mix and ROX reference dye (20×) (Qiagen, Hilden, Germany). The thermal profile was as follows: initiation (2 min, 95 °C), denaturation (10 s, 95 °C, 40 cycles), annealing/elongation (1 min, 56 °C, 40 cycles) and dissociation curve analysis. Results were analyzed using the QIAGEN GeneGlobe miRCURY LNA miRNA PCR Data analysis software. That is, C_t_ values were evaluated in consideration of an interplate calibrator, RNA isolation efficiency spike-in controls and reverse transcription efficiency spike-in control, and subsequently normalized to the expression of a set of housekeeping genes such as the miRNAs *miR-103a-3p*, *-16-5p*, *-191-5p*, *-423-5p* and *-23a-3p* and a small nucleolar RNA (*snoRD38D*) and referred to the values of the solvent control (2.5% ACN for 8 h and 0.35% ACN for 24 h), which is in accordance with the 2^−ΔΔCt^ method [40].

### 2.4. RNA Isolation and qPCR Analysis

To investigate the deregulation of gene expression of the selected liver-specific miRNAs, HepaRG cells were seeded, cultivated and adapted to treatment medium as described in Section 2.3. The cells were incubated with 35 µM Sc for 2, 4, 6, 8 and 24 h, respectively. The mRNA and miRNA were isolated using the miRNeasy Mini Kit (Qiagen, Hilden, Germany) according to the manufacturer’s protocol, including a purification step of miRNA-enriched fractions using the RNeasy MinElute Cleanup Kit (Qiagen, Hilden, Germany). Cell lysis and total RNA isolation occurred as mentioned in Section 2.3. with the exception that no spike-in control was added. Additionally, the RNeasy Mini spin columns containing the mRNA fractions were not discarded, but stored at 4 °C until further isolation. The flow-through containing the smaller miRNA fractions was purified and miRNA eluted as mentioned in Section 2.3. Afterwards, mRNA isolation occurred as follows: washing of RNeasy Mini columns once with 700 µL RWT buffer and twice with 500 µL RPE buffer with centrifugation for 15 s at 8000× *g* at RT between each step, respectively, and drying by centrifugation for 1 min at full speed at RT. The samples were eluted in 30 µL water. Both the miRNA enriched samples and the total RNA samples were quantified at 260 and 280 nm on a TecanM200Pro spectrometer (Tecan Group Lt., Männedorf, Switzerland).

Five hundred ng miRNA were polyadenylated and reverse transcribed into cDNA using the miScript II RT Kit (Qiagen, Hilden, Germany) according to the manufacturer’s protocol. The cDNA was synthesized by using a poly(T) primer with a 5′ universal tag, enabling the alignment with a universal primer during qPCR amplification. qPCR was performed from 227.3 pg cDNA on an ABI 7900HT Fast Real-Time PCR system in 384-well format using miScript SYBR Green PCR Kit (Qiagen, Hilden, Germany) with 10x universal primer and 10x miRNA-specific miScript Primer Assay (Qiagen, Hilden, Germany). The thermal profile was as follows: initial activation (15 min, 95 °C), 3-step cycling (40 cycles in total) with denaturation (15 s, 94 °C), annealing (30 s, 55 °C), elongation (34 s, 70 °C), and dissociation curve analysis. C_t_ values were evaluated according to the 2^−ΔΔCt^ method [40] by normalizing the respective C_t_-values to the expression level of miR-103a and referring to the solvent control (0.35% ACN).

One microgram of total RNA was reverse transcribed into cDNA using the High-Capacity cDNA Reverse Transcriptase Kit (Applied Biosystems, Foster City, CA, USA) according to the manufacturer’s protocol. Using 20 ng cDNA, qPCR was conducted on an ABI 7900HT Fast Real-Time PCR system in 384-well format with Maxima SYBR Green/ROX qPCR Mastermix (Thermo Fisher Scientific, Waltham, MA, USA) and 300 nM of each primer (synthesized at Eurofins Genomics, Ebersberg, Germany, see Table 1). The thermal profile was as follows: initial denaturation (15 min, 95 °C), 3-step cycling (40 cycles in total) with denaturation (30 s, 95 °C), annealing and elongation (1 min, 60 °C), final elongation (10 min, 60 °C), and dissociation curve analysis. C_t_ values were evaluated according to the 2^−ΔΔCt^ method and normalized to the housekeeping gene *GUSB* (β-glucuronidase).

### 2.5. Ingenuity Pathway Analysis

The target genes of miRNAs with significantly altered expression values in HepaRG cells after treatment with 35 µM Sc for 24 h were predicted with Ingenuity Pathway Analysis software (IPA, version 70750971, Qiagen Bioinformatics, Redwood City, CA, USA). IPA uses experimentally validated targeting interactions from TarBase [41], miRecords [42] and Ingenuity expert findings, and predicted miRNA–mRNA interactions from TargetScan [43]. The predicted target genes were compared to the gene expression dataset from a whole genome microarray conducted with primary human hepatocytes incubated with 100 µM Sc for 24 h [44]. The matched targets were prioritized according to their miRNA and mRNA relationship. This included opposing expression pairing and “highly predicted” and/or “experimentally observed” confidence of miRNA-target gene correlation. Subsequently, IPA core analysis and expression analysis with the matched target genes were performed to predict affected diseases and functions and to determine possible signaling pathways involved in PA toxicity.

### 2.6. AntagomiR Experiments

In order to identify the targets of the miRNA-4434 (also known as human miRNA-4516) and to assess if this specific miRNA expression is sensitive to PA exposure in HepaRG cells, so-called antagomiR experiments were conducted. For this purpose, HepaRG cells were seeded, cultivated and adapted to treatment medium as described in Section 2.3. The cells were incubated with 35 µM Sc and transfected with Lipofectamine RNAiMAX (Thermo Fisher Scientific Waltham, MA, USA) reagent with either miRCURY LNA miRNA Power Inhibitor (antagomiR)-4434 (5′-3′ sequence: TTCTACTTTACTTCTCCT, matches miRNA-4434 in its seed region GGAGAAG), miScript Inhibitor Negative Control (5′-3′ sequence: TAACACGTCTATACGCCCA; both from Qiagen, Hilden, Germany) as an antagomiR-Negative Control (antagomiR-NC) or water as a negative treatment control. RNAiMAX and antagomiR-4434, antagomiR-NC or water were prepared separately in Opti-MEM reduced serum medium before being mixed 1:1 and incubated for 5 min at RT. Afterwards, the antagomiR-lipid complexes were added to the cells, resulting in a final concentration of 100 pmol (50 nM) or 150 pmol (75 nM) antagomiR-4434 or anagomiR-NC per 6-well, respectively. According to the manufacturer’s protocol, antisense effects are usually assessed 24–72 h after transfection. Therefore, in this case, total RNA was isolated 48 h after transfection and isolation was conducted with the RNeasy Mini Kit (Qiagen, Hilden, Germany) according to the manufacturer’s instructions, as described elsewhere [45]. Quantity and purity of isolated RNA were measured at 260 and 280 nm on a TecanM200Pro spectrometer. Then, 1 µg total RNA was reverse transcribed into cDNA and qPCR was performed from 20 ng cDNA as described in Section 2.4.

### 2.7. Cell Viability Assay

In order to exclude antagomiR-mediated cytotoxicity on PA-incubated HepaRG cells, cells were seeded at a density of 9000 cells per well in the inner 60 wells of a 96-well plate and cultivated as described in Section 2.2. After differentiation, cells were incubated with 35 µM Sc and transfected with either antagomiR-4434 or antagomiR-NC at concentrations of 25, 50, 75 and 100 nM, respectively, as described in Section 2.5. Transfection reagent with water was used as a negative control and 0.01% Triton-X-100 served as positive control for cytotoxicity. The Neutral Red Uptake (NRU) assay was applied for assessing the cell viability. Briefly, 120 µL 3-amino-7-dimethylamino-2-methylphenazine hydrochloride (neutral red) reagent dissolved in cell culture medium (40 µg/mL) was added per well and incubated for 2 h at 37 °C. Afterwards, the supernatant was discarded, cells were washed twice with Phosphate Buffered Saline (PBS) and dye crystals dissolved in 150 µL desorption solution (1% acetic acid in 50% ethanol). After shaking for 10 min (under protection from light), fluorescence was measured at λ_ex_ = 530 nm and λ_em_ = 645 nm on a TecanM200Pro spectrometer.

### 2.8. Statistical Analysis

Statistical analysis was performed using SigmaPlot 14.0 software (Systat Software, Erkrath, Germany). All qPCR data were subjected to logarithmic tranformation and statistically significant differences were calculated by Student’s *t*-test in the case of single comparison analyses. If applicable, *p*-values were subjected to False Discovery Rate (FDR) correction [46]. Results were considered as significant at *p* < 0.05 and are indicated in the graphs by * *p* < 0.05, ** *p* < 0.01, *** *p* < 0.001.

## 3. Results

### 3.1. Sc Induces miRNA Expression in HepaRG Cells

**Figure 1:**
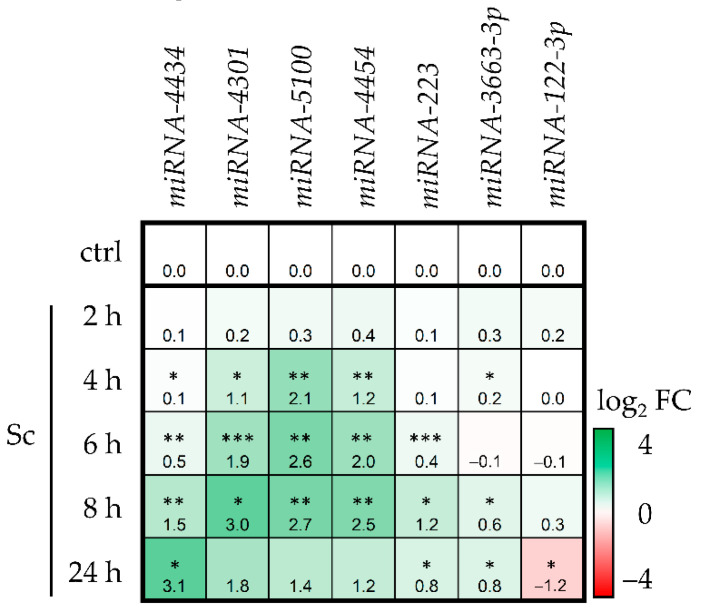
miRNA expression in differentiated HepaRG cells after exposure to 35 µM Sc at 5 different time-points. The results were evaluated using the 2^−∆∆Ct^ method [40]. Results are shown as log_2_ fold changes (log_2_ FC) and as mean of three independent biological replicates. Gene expression values were referred to the solvent control (ctrl) of the respective time point but, for clarity, only the ctrl values of the 2 h time point are depicted here. Up- or downregulation of gene expression is indicated in green or red, respectively. The higher the values, the stronger the coloration. Mean values and standard deviations (SD) are summarized in the Supplementary Materials Table S2. Statistical analysis was performed using Student’s *t*-test as this is a case of single comparison analysis. That is, each miRNA expression value of one time point was compared to the ctrl of this respective time point. Subsequently, *p*-values were subjected to FDR correction. Significant differences are depicted as follows: * *p* < 0.05, ** *p* < 0.01, *** *p*< 0.001.

First, the miRNA expression profile in HepaRG cells was analyzed with a customized liver-specific miCURY LNA Array covering 84 miRNAs. Compared to the control group, the number of deregulated miRNAs above 1 log_2_ Fold Change (FC) or below 1 log_2_ FC tended to be highest at 250 µM after 8 h and at 35 µM after 24 h, respectively (see Supplementary Figure S1). Among the top deregulated miRNAs, a selection of five downregulated and four upregulated miRNAs were chosen to be investigated within senecionine (Sc)-treated HepaRG cells. Sc was used for qPCR in order to have greater certainty for general PA effects and to exclude very specific effects of one single PA. To this end, HepaRG cells were treated with 35 µM of Sc and miRNA expression was analyzed after five different time points. Seven out of nine miRNAs proved to be differentially expressed after Sc treatment. The results are summarized in a heat map in Figure 1. Interestingly, all but one miRNA showed an increased expression compared to the solvent control in a time-dependent manner and for three miRNAs, the upregulation was highest after 8 h. *miRNA-122-3p* (alias miRNA-122*) was the only one which showed significant downregulation after 24 h.

### 3.2. Prediction of Biological Consequences of PA-Mediated miRNA Level Alterations

**Figure 2:**
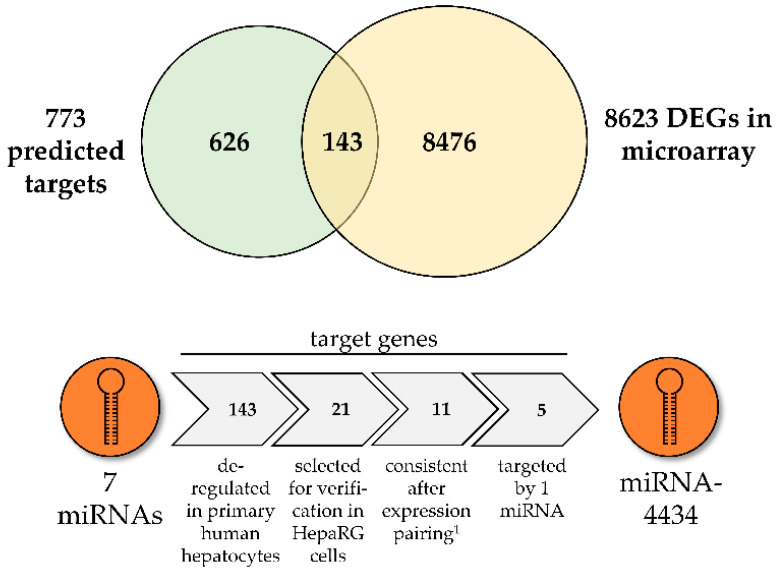
Workflow of miRNA target gene prediction with IPA. The target genes of 7 differentially and significantly expressed miRNAs in HepaRG cells were predicted with IPA (773 predicted targets) and compared to the dataset of a whole genome microarray conducted with primary human hepatocytes [44]. Among the 8623 Differentially Expressed Genes (DEGs) of the primary human hepatocytes after Sc-treatment (100 µM, 24 h), 143 targets were found to overlap with the predicted miRNA target genes. Out of these, 21 targets that simultaneously showed both a significant deregulation in the microarray as well as a high (predicted) confidence of miRNA-target interaction, were selected to be further investigated in HepaRG cells exposed to 35 µM of Sc for 5 different time points. A total of 11 out of 21 target genes showed an opposing expression pairing to their annotated miRNAs (indicated by superscript number 1), as verified by qPCR in HepaRG cells. IPA analysis revealed that 5 out of 11 targets were regulated by one miRNA (miRNA-4434).

Subsequently, the target genes of seven miRNAs that were identified to have significantly altered expression log_2_ FC values in HepaRG cells (see Figure 1) were predicted with IPA software (see Figure 2 for workflow).

**Figure 3:**
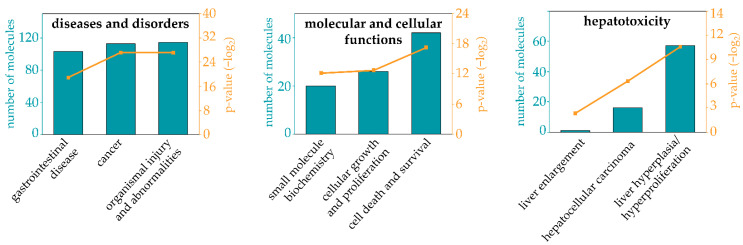
Top 3 pathways in 3 “diseases and bio functions” of deregulated target genes in primary human hepatocytes listed in IPA after expression analysis with 7 miRNAs. IPA target gene prediction of 7 significantly deregulated miRNAs in HepaRG cells treated with 35 µM of Sc for 24 h was compared to the gene expression data from Sc-treated primary human hepatocytes (100 µM, 24 h) [44]. The numbers of deregulated target genes (number of molecules) predicted to be involved in diseases and bio functions are depicted as bar charts and their lower range of the *p*-value is shown in −log_2_ FC (orange graph).

The predicted target genes (773 in total) were compared to the gene expression dataset from a whole genome microarray conducted with primary human hepatocytes incubated with 100 µM Sc for 24 h [44]. Matched and prioritized targets (143 target genes) were submitted to a so-called IPA expression analysis to predict affected diseases and functions. In Figure 3 the top “diseases and bio functions” of the deregulated target genes of seven selected miRNAs predicted in Sc-treated primary human hepatocytes are shown. “Cancer” and “organismal injury and abnormalities” are the two main diseases and disorders that are regulated by the highest number of predicted targets. Among molecular and cellular functions, “cell death and survival” has the highest number of target genes with the most pronounced statistical significance. “Liver hyperproliferation” is the top hepatotoxicity function with 57 target genes involved, followed by “hepatocellular carcinoma” with 16 target genes.

**Figure 4:**
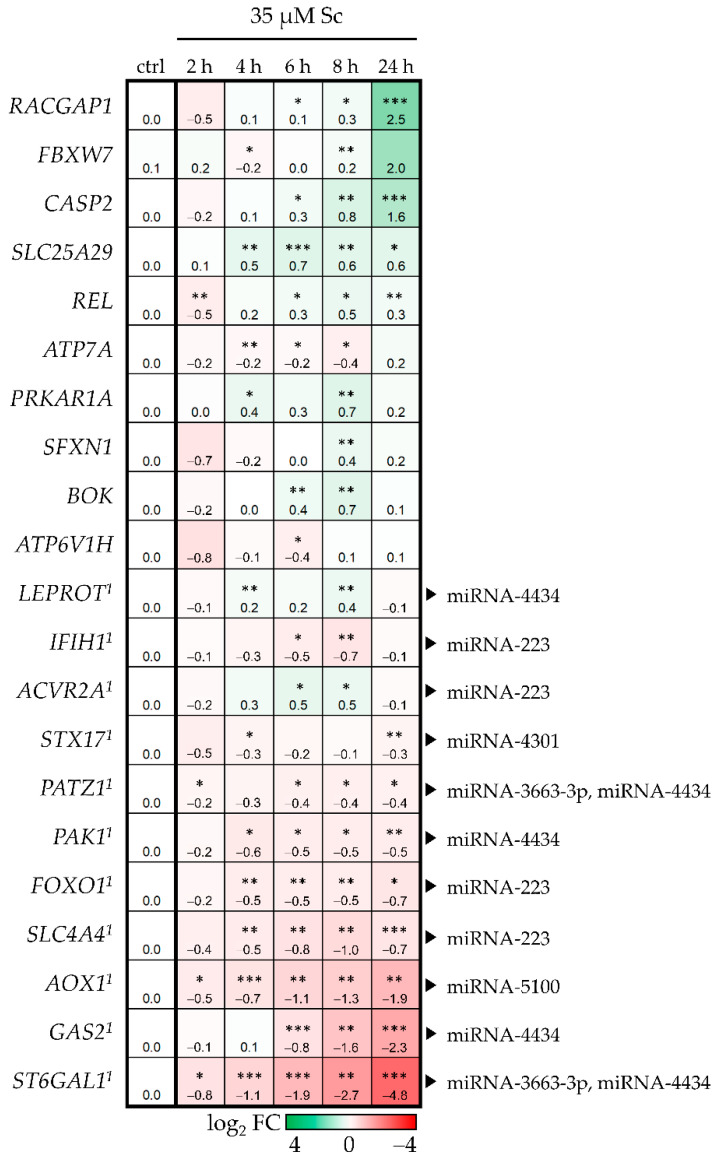
Target gene expression in differentiated HepaRG cells after exposure to 35 µM Sc at 5 different time points. The results were evaluated using the 2^−∆∆Ct^ method [40]. Results are shown as log_2_ FC and as mean of three biological replicates. Gene expression values were referred to the solvent control (ctrl) of the respective time point but, for clarity, only the ctrl values of the 2 h time point are depicted here. Up- or downregulation of gene expression is indicated in green or red, respectively. The higher the values, the stronger the coloration. Mean values and standard deviations are summarized in the Supplementary Materials Table S3. Superscript number 1 indicates the target genes that show an opposite regulation to their annotated miRNAs and the black arrows indicate the predicted regulatory miRNAs, respectively. Statistical analysis was performed using Student’s *t*-test as this is a case of single-comparison analysis. That is, each target gene expression value of one time point was compared to the solvent control of this respective time point. Subsequently, *p*-values were subjected to FDR correction. Significant differences are depicted as follows: * *p* < 0.05, ** *p* < 0.01, *** *p*< 0.001.

Based on the expression analysis, 21 target genes involved in cellular growth and development, apoptosis and inflammation were chosen to be further investigated by qPCR in HepaRG cells incubated with 35 µM Sc for five different time points. The results are depicted in Figure 4 as a heat map. A total of 11 out of 21 genes showed an opposing expression pairing (indicated by superscript number 1); that is, significant downregulation, while their attributed miRNAs were upregulated. For the miRNA-4434 in particular, interaction with 5 out of 11 targets was predicted. These five targets comprised Growth Arrest-Specific protein 2 (*GAS2*), P21-Activated Kinase-1 (*PAK1*), LEPtin Receptor Overlapping Transcript (*LEPROT*), POZ/BTB and AT hook containing Zinc finger 1 (*PATZ1*) and ST6 beta-GALactoside alpha-2,6-sialyltransferase 1 (*ST6GAL1*). Therefore, this miRNA was chosen for further experiments.

### 3.3. MiR-4434 Negatively Regulates PAK1 Gene Expression in HepaRG Cells

**Figure 5:**
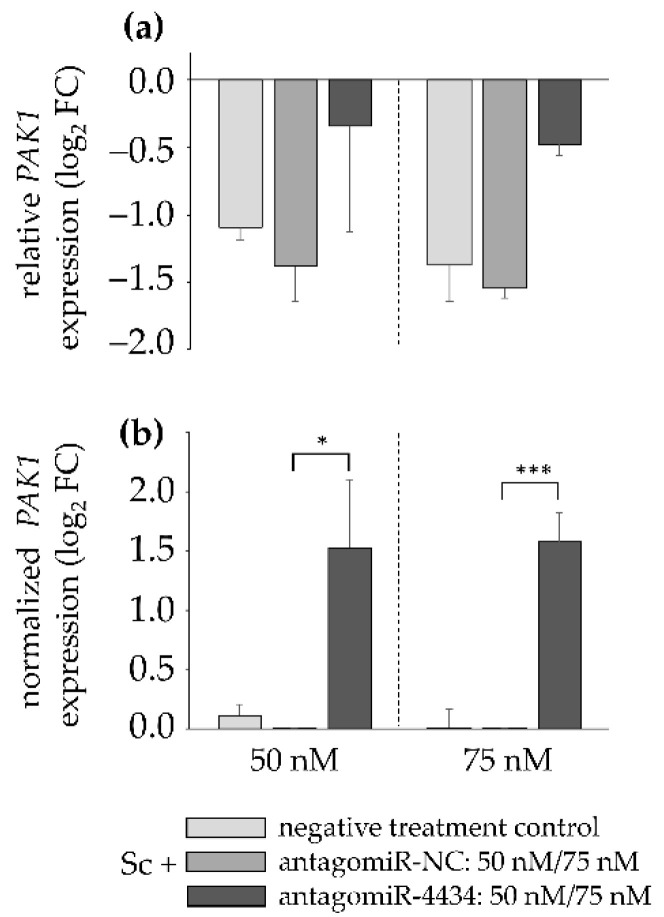
*PAK1* gene expression in differentiated HepaRG cells after exposure to 35 µM Sc and antagomiR-mediated inhibition of miR-4434 after 48 h. The results were evaluated according to the 2^−∆∆Ct^ method [40]. (**a**) After housekeeper normalization, the values of the Sc-treated cells were referred to the respective solvent control (0.35% ACN). Noteworthy, the different treatments (antagomiR-4434, antagomiR-NC and negative treatment control) were referred to their respective solvent controls. AntagomiR/antagomiR-NC treatment was applied in the two concentrations 50 and 75 nM. Results are shown as log_2_ FC and as mean of three replicates. Mean values and standard deviations are summarized in the Supplementary Materials Table S4. (**b**) For normalized *PAK1* expression analysis within the Sc-treated cells, antagomiR-4434 treatment was referred to antagomiR-NC treatment. Again, antagomiR/antagomiR-NC treatment was applied in the two concentrations 50 and 75 nM. Additionally, the negative treatment control was referred to antagomiR-NC to ensure specific antagomiR-mediated inhibition. Statistical analysis was performed using Student’s *t*-test as this is a case of single-comparison analysis. That is, the value of *PAK1* gene expression with antagomiR-4434 treatment (50 and 75 nM, respectively) was compared to the value of *PAK*1 gene expression with antagomiR-NC treatment (50 and 75 nM, respectively). Significant differences are depicted as follows: * *p* < 0.05, *** *p* < 0.001.

In order to investigate whether it is indeed the PA-mediated induction of miR-4434 levels that leads to the downregulation of the pre-selected target genes, differentiated HepaRG cells were treated with 35 µM of the PA Sc and, subsequently, transiently transfected either with the synthetic miRNA antagonist antagomiR-4434 or with an antagomiR-NC (both at 50 and 75 nM concentrations) or with transfection reagent and water only, as a negative treatment control. The miRNA inhibitor does not degrade its target, but forms a stable complex with it, resulting in an inhibition of miRNA-4434 function. The antagomiR-NC is non-homologous to any mammalian gene and was used to examine if the results of the antagomiR-4434-mediated inhibition were specific. That is, results achieved with the antagomiR-NC should be similar to results from negative treatment control (transfection reagent and water only). It was ensured that the combined incubation with antagomiR and Sc was non-cytotoxic (see Supplementary Materials Figure S2). The gene expression of all 21 target genes was assessed after 48 h and the values of the Sc-treated cells were always referred to their respective solvent control (antagomiR-4434, antagomiR-NC, or negative treatment control) (see Supplementary Materials Figure S3). When comparing antagomiR-4434-transfected cells with the negative control (antagomiR-NC) within the Sc-treatment group, the antisense effect of the antagomiR and its subsequent effect on miR-4434 target gene expression becomes evident. Here, the inhibition of miR-4434 led to significantly attenuated downregulation of *PAK*1 gene expression, with increased significance at the higher antagomiR concentration (Figure 5).

## 4. Discussion

This study aimed to investigate the PA-induced effects on miRNA expression. Furthermore, miRNA-mediated effects on potential target genes were considered as an additional, however indirect, molecular regulator in PA-mediated hepatotoxicity. In the last years, miRNAs have emerged as very important players in the regulation of a variety of biological processes in many cell types, including those in the liver [32]. miRNA function ensures fine tuning of target gene expression, and subsequently protein abundance and protein distribution under constantly changing cellular conditions [47]. For example, in the liver intracellular miRNA levels regulate lipid and glucose metabolism [48], inflammation [49,50,51], apoptosis and necrosis [52,53,54], cell cycle and proliferation [55], as well as epithelial–mesenchymal transition during liver regeneration [56] and liver fibrosis [57,58]. Alterations in physiological miRNA levels correlate with various liver diseases such as viral hepatitis, alcoholic and nonalcoholic steatohepatitis, drug-induced liver injury, and autoimmune liver disease [32]. In metazoan cells, it is generally assumed that translational repression, presumably occurring during translation initiation, is the predominant mechanism by which miRNAs negatively regulate their target genes [29]. Therefore, it could be concluded that protein analysis is the investigative method of choice. However, transcript degradation of the target mRNA is an unfailing secondary effect triggered by the initial event of translational repression [59,60,61]. Therefore, qPCR analysis of target mRNA expression is a reliable and widely used method to assess the effects of miRNA-mediated gene silencing and was also applied here.

To elucidate the regulatory effects of miRNAs on their target genes is rather challenging because of its sheer complexity. For example, one miRNA can regulate many targets, and one target can be regulated by many different miRNAs in turn. Furthermore, miRNAs can directly bind their target gene, or they can indirectly regulate a target gene via binding to regulatory molecules like transcription factors, which have their own mode of action [62]. Thus, miRNAs can actually decrease, increase or not change the expression levels of their target genes [63]. This study aimed to achieve the first insights into the mechanistic of miRNA expression in HepaRG cells to understand its implication in PA-mediated hepatotoxicity. Therefore, for simplicity we only considered target genes that showed reverse correlation in gene expression in relation to their assigned miRNAs. Thus, target genes potentially showing a positive correlation or no correlation at all could have been overlooked. In general, miRNA upregulation upon PA-treatment has been described before. For example, *miRNA-34a*, which is considered to be a biomarker for exposure to genotoxic compounds, showed significant upregulation after chronic PA ingestion in a feeding study with rats [35]. Furthermore, blood samples from patients with HSOS showed elevated levels of miRNA-148a-3p, miRNA-362-5p, and miRNA-194-5p, which could be correlated to the severity of the PA-induced liver injury [37].

Upon Sc-incubation, seven liver-specific miRNAs could be verified to be significantly deregulated in HepaRG cells. All but one showed increased gene expression in a time-dependent manner. For *miRNA-4301, miRNA-5100* and *miRNA-4454*, upregulation was highest after 8 h, showing the early responsiveness of miRNA levels. miRNA-4301 and miRNA-5100 have been observed to regulate proliferation and apoptosis in lung and breast cancer cells [64,65,66,67], but according to our assessment there are no reports on their implication in hepatic diseases. For *miRNA-4454*, it was reported that upregulation positively enhances hepatic carcinoma progression [68], miR-223 is a common regulator in various liver diseases [69], and *miRNA-3663-3p* was shown to be downregulated in hepatocellular carcinoma cells, thus positively regulating cell proliferation of cancer cells [70]. The involvement of these three miRNAs in liver diseases might pose an interface between their PA-induced upregulation and possible carcinogenic properties that have been described for PAs [71,72,73]. *miRNA-4434* had the highest upregulation of all miRNAs investigated. This miRNA plays a role in different tumors, either as promoter or inhibitor of proliferation [74,75,76]. However, reports on its relevance in liver function and disease are rare. One study observed miRNA-4434 to be inhibited by a long non-coding RNA (lncRNA) called Long Stress Induced Non-Coding Transcripts 5 (LSINCT5) in hepatocellular carcinoma progression, potentially resulting in inhibited miRNA-4434 induced apoptosis [77]. *miRNA-122-*3p (alias miRNA-122*, derived from the antisense strand of the precursor (pre)-miRNA; [78]) was the only miRNA that showed a significant downregulation only after 24 h. Noteworthy, *miRNA-122-*3p downregulation has been observed during early and advanced liver fibrosis [79]. As fibrosis is a disease which is also observed after chronic PA-intoxication, this could point towards a connection between downregulated *miRNA-122-3p* levels and PA-mediated toxicity resulting in fibrosis. The most abundant miRNA in the liver, *miRNA-122-5p* (alias miRNA-122a, derived from the sense strand of the pre-miRNA), did not show a statistically relevant deregulation in gene expression after Sc-treatment at any time-point and was therefore excluded from further analysis.

The comparison analysis between IPA-predicted mRNA targets and differentially expressed genes obtained from a whole genome microarray [44] revealed a target match of 143 genes that are involved in many diseases and disorders such as cancer in general and hepatocellular carcinoma in particular, with cellular growth and proliferation as underlying molecular and cellular functions. Of course, this analysis is a rather generalized evaluation. Therefore, out of these targets, a set of 21 genes regulating processes in cellular growth, development, apoptosis and inflammation was chosen to be verified in Sc-treated HepaRG cells and subjected to further IPA analysis. For cAMP-dependent PRotein Kinase type I-Alpha Regulatory subunit (*PRKAR1A*), a miRNA-dependent regulation was also observed in Mc-treated mice for type II-alpha subunit (*Prkar2a*). Moreover, in the same study, target gene V-type proton ATPase subunit e 2 (*Atp6v0e2*) also showed a significant upregulation in a miRNA-dependent way [36]. Here, another subunit type (*ATP6V1H*) was selected. Both *PRKAR1A* and *ATP6V1H* showed upregulation of gene expression, as was observed in the study mentioned above. A small subgroup of 11 genes showed reverse correlation in gene expression in comparison to their assigned miRNAs. These five miRNAs out of the initial set of seven were miRNA-223, miRNA-3663-3p, miRNA-4301, miRNA-4434, and miRNA-5100. According to IPA analysis, the 11 dysregulated genes generally promote apoptosis and necrosis and decrease cellular survival. Interestingly, miRNA-4434 was predicted to be an upstream regulator of 5 out of the 11 genes, which was the highest number of annotated targets for one miRNA. Surprisingly, it was not a well-established hepatic miRNA such as miRNA-223 or miRNA-122-5p, but the rather less-known miRNA-4434. The five target genes are involved in processes such as cell cycle progression (*GAS2* and *PAK1*), growth hormone signaling (*LEPROT*), apoptosis (*PATZ1*) and immunity (*ST6GAL1*), suggesting a correlation between PA-induced upregulation of miRNAs and subsequent downregulation of these five target genes, presumably resulting in disturbed cellular function. Ultimately, antagomiR-mediated inhibition of miRNA-4434 resulted in significantly altered gene expression pattern of the target gene *PAK*1, strongly indicating a biological connection. The *PAK1* gene encodes one family member of the serine/threonine-specific intracellular protein kinases that are involved in a number of cellular functions including cell cycle regulation, apoptosis, and cytoskeletal motility, usually through substrate phosphorylation [80]. PAK1 is a regulator in key signaling pathways which are relevant for cell cycle progression and proliferation. On the one hand, *PAK1* is overexpressed in many cancers and positively correlates with promotion of cell survival, invasion and metastasis, and drug resistance [81]. In the context of tumor therapy, for example, PAK1 triggers DNA repair caused by genotoxic therapeutic agents [82]. On the other hand, *PAK1* hinders cell cycle progression upon inhibition [83]. Additionally, miRNA interaction with *PAK1* expression has been described previously: in hepatocellular carcinoma development, miRNA-485-5p was observed to suppress *PAK1* levels, and lncRNA-mediated binding of miRNA-485-5p resulted in the upregulation of *PAK1* during hepatocellular carcinoma progression [84]. Here, a PA-induced upregulation of miRNA-4434 can be assumed to negatively regulate *PAK1* expression, presumably resulting in cell-cycle arrest. Notably, an implication of PAs in cell cycle regulation, which is closely linked to DNA damage, has been observed before [85]. This effect was observed to be higher for the more toxic PAs in comparison to the less toxic PAs. In conclusion, we suggest that the identification of the regulatory mechanism of miRNA-4434-initiated and *PAK1*-induced dysregulation of cell cycle signaling may help to understand the molecular mode of action of some hepatotoxic and carcinogenic effects of Sc.

## Figures and Tables

**Table 1:** Sequences of primers used for qPCR analysis of target genes.

Gene	Ensembl ID	Forward Primer (5′–3′)	Reverse Primer (5′–3′)
*ACVR2A*	ENSG00000121989	TGTTTTGGGCACAGGTTATTGT	CAAGGTGGGGTTTTATGGGGA
*AOX1*	ENSG00000138356	TCCCTGCCATCTGTGACATG	CCGACTCTCCCAGACCCTTA
*ATP6V1H*	ENSG00000047249	CCAAAACTGTGGCCATGCTA	TGCTGGTGAGAGGCTTCAGA
*ATP7A*	ENSG00000165240	CAAAACCAGGCGTCTCAACC	TCGTCCACTGCTGTTTTCGG
*BOK*	ENSG00000176720	CACACACAGCCTTCCCTTGA	GCCTGTATCTCCTGAGTGCC
*CASP2*	ENSG00000106144	GAATTCCACCGGTGCAAGGA	TGGATGATGGGGAGGTGACA
*FBXW7*	ENSG00000109670	TATACTCCCTGCCCTTCCCC	CCAACATCCTGCACCACTGA
*FOXO1*	ENSG00000150907	CAGGGGTGGCCATGTAAGTC	GGAACAAGAACGTGGAATCTGC
*GAS2*	ENSG00000148935	GTGCCGAGATTTAGGGGTGG	GCAATCCGGCCAAGCTCTAG
*GUSB*	ENSG00000169919	CACCAGGGACCATCCAATAC	ATGTAGGTGGTGGGTGTCGT
*IFIH1*	ENSG00000115267	CAAAAGAAGTGTGCCGACTATCA	TGCACCATCATTGTTCCCCA
*LEPROT*	ENSG00000213625	ATTGAGTTCCCAGGCCAAGC	AGACCCAAAACTCAGGCAGG
*PAK1*	ENSG00000149269	GCCTGACATGATACCCTGCC	AGCAAACATCCCCAACACCC
*PATZ1*	ENSG00000100105	AGTCTGGTCAGGGAAGTAGGG	ACACAATGTCCCTACCTGCC
*PRKAR1A*	ENSG00000108946	ACTTTGCTGGAGTGGTGGTG	TGGTCTTGAACTCCTGGGCT
*RACGAP1*	ENSG00000161800	CTGTCCCCTTCCCTGCATTC	TGCACAACAATGGAGGGGAT
*REL*	ENSG00000162924	CTTCATGCCCCTTCCCAGTC	ACGTTGACAACCCAGCTGTT
*SFXN1*	ENSG00000164466	AACAGGGTCATGCTTGGATCA	GGCTTCTTGAGGTCTCTGGC
*SLC25A29*	ENSG00000197119	GCCCACACTGTAGAGTCACG	AGAAAGGGGCTGGAGTGTCT
*SLC4A4*	ENSG00000080493	CAAACATTGCAACTCAGGGCT	TTCACATTGTAGGACTGGGACA
*STX17*	ENSG00000136874	TGTTAGCAAGGGTCAGCACG	TCAAGCACCACTCAGCAATGT
*ST6GAL1*	ENSG00000073849	AGCCCACTTTCCCTCTCCAT	TGCCTCTCTCACTGAACCGT

## Data Availability

Data is contained within the article (or Supplementary Material).

## Supplementary Materials

The following supporting information can be downloaded at: https://www.mdpi.com/article/10.3390/foods11040532/s1: Table S1: miRNAs investigated with miRCURY LNA miRNA custom PCR array, including 84 liver-specific miRNAs, 7 housekeeping genes, 1 interplate calibrator, 3 RNA isolation efficiency spike-in controls and 1 reverse transcription efficiency spike-in control; Figure S1: miRNA expression profile in differentiated HepaRG cells after exposure to Lc and determined with miRCURY LNA miRNA Array; Table S2: miRNA expression in differentiated HepaRG cells after exposure to 35 µM Sc at 5 different time-points; Table S3: Target gene expression in differentiated HepaRG cells after exposure to 35 µM of Sc for 5 time points; Figure S2: Cytotoxicity of differentiated HepaRG cells after antagomiR/antagomiR-NC- and Sc-incubation; Figure S3: Target gene expression in differentiated HepaRG cells after exposure to 35 µM Sc and antagomiR-mediated inhibition of miR-4434 after 48 h; Table S4: Target gene expression in differentiated HepaRG cells after exposure to 35 µM Sc and antagomiR-mediated inhibition of miR-4434 after 48 h.

## Abbreviations

ALARA	As Low As Reasonably Achievable
*ATP6V1H/0E2*	V-type proton ATPase subunit H/E 2
DEG	Differentially Expressed Gene
DMSO	DiMethyl SulfOxide
FBS	Fetal Bovine Serum
FC	Fold Change
FDR	False Discovery Rate
*GAS2*	Growth Arrest-Specific protein 2
*GUSB*	β-glucuronidase
HSOS	Hepatic Sinusoidal Obstruction Syndrome
IPA	Ingenuity Pathway Analysis
Lc	Lasiocarpine
lncRNA	long non-coding RNA
*LSINCT5*	Long Stress-Induced Non-Coding Transcripts 5
*LEPROT*	LEPtin Receptor Overlapping Transcript
Mc	Monocrotaline
miRNA	microRNA
NRU	Neutral Red Uptake
PA	1,2-unsaturated PyrrolizidineAalkaloid
*PAK1*	P21-Activated Kinase-1
*PATZ1*	POZ/BTB and AT hook containing Zinc finger 1
*PRKAR1A/2A*	cAMP-dependent PRotein Kinase type I (II)-Alpha Regulatory subunit
PXR	Pregnane X Receptor
qPCR	quantitative real-time Polymerase Chain Reaction
RT	Room Temperature
Sc	Senecionine
*ST6GAL1*	ST6 beta-GALactoside alpha-2,6-sialyltransferase 1
TDI	Tolerable Daily Intake
UTR	3′-UnTranslated Region

## References

[1] Mattocks A. (1986). Chemistry and Toxicology of Pyrrolizidine Alkaloids.

[2] Mulder P.P.J., López P., Castellari M., Bodi D., Ronczka S., Preiss-Weigert A., These A. (2018). Occurrence of pyrrolizidine alkaloids in animal- and plant-derived food: Results of a survey across Europe. Food Addit. Contam. Part A Chem. Anal. Control Expo. Risk Assess..

[3] Bodi D., Ronczka S., Gottschalk C., Behr N., Skibba A., Wagner M., Lahrssen-Wiederholt M., Preiss-Weigert A., These A. (2014). Determination of pyrrolizidine alkaloids in tea, herbal drugs and honey. Food Addit. Contam. Part A Chem. Anal. Control Expo. Risk Assess..

[4] Edgar J.A., Lin H.J., Kumana C.R., Ng M.M. (1992). Pyrrolizidine alkaloid composition of three Chinese medicinal herbs, *Eupatorium cannabinum*, *E. japonicum* and *Crotalaria assamica*. Am. J. Chin. Med..

[5] Kaltner F., Rychlik M., Gareis M., Gottschalk C. (2020). Occurrence and Risk Assessment of Pyrrolizidine Alkaloids in Spices and Culinary Herbs from Various Geographical Origins. Toxins.

[6] BfR (2013). Pyrrolizidine Alkaloids in Herbal Teas and Teas.

[7] Ruan J., Yang M., Fu P., Ye Y., Lin G. (2014). Metabolic activation of pyrrolizidine alkaloids: Insights into the structural and enzymatic basis. Chem. Res. Toxicol..

[8] Bach N., Thung S.N., Schaffner F. (1989). Comfrey herb tea-induced hepatic veno-occlusive disease. Am. J. Med..

[9] Ridker P.M., McDermott W.V. (1989). Comfrey herb tea and hepatic veno-occlusive disease. Lancet.

[10] Ridker P.M., Ohkuma S., McDermott W.V., Trey C., Huxtable R.J. (1985). Hepatic venocclusive disease associated with the consumption of pyrrolizidine-containing dietary supplements. Gastroenterology.

[11] Kumana C.R., Ng M., Lin H.J., Ko W., Wu P.C., Todd D. (1985). Herbal tea induced hepatic veno-occlusive disease: Quantification of toxic alkaloid exposure in adults. Gut.

[12] Roitman J.N. (1981). Comfrey and liver damage. Lancet.

[13] Edgar J.A., Molyneux R.J., Colegate S.M. (2015). Pyrrolizidine Alkaloids: Potential Role in the Etiology of Cancers, Pulmonary Hypertension, Congenital Anomalies, and Liver Disease. Chem. Res. Toxicol..

[14] Edgar J.A., Roeder E., Molyneux R.J. (2002). Honey from plants containing pyrrolizidine alkaloids: A potential threat to health. J. Agric. Food Chem..

[15] Svoboda D., Reddy J., Bunyaratvej S. (1971). Hepatic megalocytosis in chronic lasiocarpine poisoning. Some functional studies. Am. J. Pathol.

[16] Jago M.V. (1969). The development of the hepatic megalocytosis of chronic pyrrolizidine alkaloid poisoning. Am. J. Pathol..

[17] Molteni A., Ward W.F., Ts’Ao C.-H., Port C.D., Solliday N.H. (1984). Monocrotaline-lnduced Pulmonary Endothelial Dysfunction in Rats. Proc. Soc. Exp. Biol. Med..

[18] Stegelmeier B.L., Edgar J.A., Colegate S.M., Gardner D.R., Schoch T.K., Coulombe R.A., Molyneux R.J. (1999). Pyrrolizidine alkaloid plants, metabolism and toxicity. J. Nat. Toxins.

[19] Waizenegger J., Braeuning A., Templin M., Lampen A., Hessel-Pras S. (2018). Structure-dependent induction of apoptosis by hepatotoxic pyrrolizidine alkaloids in the human hepatoma cell line HepaRG: Single versus repeated exposure. Food Chem. Toxicol..

[20] Ebmeyer J., Braeuning A., Glatt H., These A., Hessel-Pras S., Lampen A. (2019). Human CYP3A4-mediated toxification of the pyrrolizidine alkaloid lasiocarpine. Food Chem. Toxicol..

[21] Glück J., Waizenegger J., Braeuning A., Hessel-Pras S. (2021). Pyrrolizidine Alkaloids Induce Cell Death in Human HepaRG Cells in a Structure-Dependent Manner. Int. J. Mol. Sci..

[22] Allemang A., Mahony C., Lester C., Pfuhler S. (2018). Relative potency of fifteen pyrrolizidine alkaloids to induce DNA damage as measured by micronucleus induction in HepaRG human liver cells. Food Chem. Toxicol..

[23] Glück J., Henricsson M., Braeuning A., Hessel-Pras S. (2021). The Food Contaminants Pyrrolizidine Alkaloids Disturb Bile Acid Homeostasis Structure-Dependently in the Human Hepatoma Cell Line HepaRG. Foods.

[24] Waizenegger J., Glück J., Henricsson M., Luckert C., Braeuning A., Hessel-Pras S. (2021). Pyrrolizidine Alkaloids Disturb Bile Acid Homeostasis in the Human Hepatoma Cell Line HepaRG. Foods.

[25] Hessel-Pras S., Braeuning A., Guenther G., Adawy A., Enge A.-M., Ebmeyer J., Henderson C.J., Hengstler J.G., Lampen A., Reif R. (2020). The pyrrolizidine alkaloid senecionine induces CYP-dependent destruction of sinusoidal endothelial cells and cholestasis in mice. Arch. Toxicol..

[26] Luckert C., Braeuning A., Lampen A., Hessel-Pras S. (2018). PXR: Structure-specific activation by hepatotoxic pyrrolizidine alkaloids. Chem. Biol. Interact..

[27] He L., Hannon G.J. (2004). MicroRNAs: Small RNAs with a big role in gene regulation. Nat. Rev. Genet..

[28] Takeuchi M., Oda S., Tsuneyama K., Yokoi T. (2018). Comprehensive analysis of serum microRNAs in hepatic sinusoidal obstruction syndrome (SOS) in rats: Implication as early phase biomarkers for SOS. Arch. Toxicol..

[29] Bartel D.P. (2004). MicroRNAs: Genomics, biogenesis, mechanism, and function. Cell.

[30] Munoz-Garrido P., García-Fernández de Barrena M., Hijona E., Carracedo M., Marín J.J.G., Bujanda L., Banales J.M. (2012). MicroRNAs in biliary diseases. World J. Gastroenterol..

[31] Garzon R., Marcucci G. (2012). Potential of microRNAs for cancer diagnostics, prognostication and therapy. Curr. Opin. Oncol..

[32] Szabo G., Bala S. (2013). MicroRNAs in liver disease. Nat. Rev. Gastroenterol. Hepatol..

[33] Marin J.J., Bujanda L., Banales J.M. (2014). MicroRNAs and cholestatic liver diseases. Curr. Opin. Gastroenterol..

[34] Song K.H., Li T., Owsley E., Chiang J.Y. (2010). A putative role of micro RNA in regulation of cholesterol 7alpha-hydroxylase expression in human hepatocytes. J. Lipid Res..

[35] Chen T., Li Z., Yan J., Yang X., Salminen W. (2012). MicroRNA expression profiles distinguish the carcinogenic effects of riddelliine in rat liver. Mutagenesis.

[36] Huang Z., Chen M., Zhang J., Sheng Y., Ji L. (2017). Integrative analysis of hepatic microRNA and mRNA to identify potential biological pathways associated with monocrotaline-induced liver injury in mice. Toxicol. Appl. Pharmacol..

[37] Wang X., Zhang W., Yang Y., Chen Y., Zhuge Y., Xiong A., Yang L., Wang Z. (2021). Blood microRNA Signatures Serve as Potential Diagnostic Biomarkers for Hepatic Sinusoidal Obstruction Syndrome Caused by *Gynura japonica* Containing Pyrrolizidine Alkaloids. Front. Pharmacol..

[38] Oda S., Takeuchi M., Akai S., Shirai Y., Tsuneyama K., Yokoi T. (2018). miRNA in Rat Liver Sinusoidal Endothelial Cells and Hepatocytes and Application to Circulating Biomarkers that Discern Pathogenesis of Liver Injuries. Am. J. Pathol..

[39] Gripon P., Rumin S., Urban S., Le Seyec J., Glaise D., Cannie I., Guyomard C., Lucas J., Trepo C., Guguen-Guillouzo C. (2002). Infection of a human hepatoma cell line by hepatitis B virus. Proc. Natl. Acad. Sci. USA.

[40] Livak K.J., Schmittgen T.D. (2001). Analysis of relative gene expression data using real-time quantitative PCR and the 2^−ΔΔCT^ Method. Methods.

[41] Karagkouni D., Paraskevopoulou M.D., Chatzopoulos S., Vlachos I.S., Tastsoglou S., Kanellos I., Papadimitriou D., Kavakiotis I., Maniou S., Skoufos G. (2018). DIANA-TarBase v8: A decade-long collection of experimentally supported miRNA–gene interactions. Nucleic Acids Res..

[42] Xiao F., Zuo Z., Cai G., Kang S., Gao X., Li T. (2009). miRecords: An integrated resource for microRNA–target interactions. Nucleic Acids Res..

[43] Agarwal V., Bell G.W., Nam J.-W., Bartel D.P. (2015). Predicting effective microRNA target sites in mammalian mRNAs. eLife.

[44] Luckert C., Hessel S., Lenze D., Lampen A. (2015). Disturbance of gene expression in primary human hepatocytes by hepatotoxic pyrrolizidine alkaloids: A whole genome transcriptome analysis. Toxicol. Vitr..

[45] Enge A.M., Kaltner F., Gottschalk C., Kin A., Kirstgen M., Geyer J., These A., Hammer H., Pötz O., Braeuning A. (2021). Organic Cation Transporter I and Na^+^ /taurocholate Co-Transporting Polypeptide are Involved in Retrorsine- and Senecionine-Induced Hepatotoxicity in HepaRG cells. Mol. Nutr. Food Res..

[46] Benjamini Y., Hochberg Y. (1995). Controlling the False Discovery Rate: A Practical and Powerful Approach to Multiple Testing. J. R. Stat. Soc. Ser. B.

[47] Bartel D.P. (2009). MicroRNAs: Target recognition and regulatory functions. Cell.

[48] Rottiers V., Näär A.M. (2012). MicroRNAs in metabolism and metabolic disorders. Nat. Rev. Mol. Cell Biol..

[49] O’Neill L.A., Sheedy F.J., McCoy C.E. (2011). MicroRNAs: The fine-tuners of Toll-like receptor signalling. Nat. Rev. Immunol..

[50] Bala S., Szabo G. (2012). MicroRNA Signature in Alcoholic Liver Disease. Int. J. Hepatol..

[51] Thai T.H., Calado D.P., Casola S., Ansel K.M., Xiao C., Xue Y., Murphy A., Frendewey D., Valenzuela D., Kutok J.L. (2007). Regulation of the germinal center response by microRNA-155. Science.

[52] An F., Gong B., Wang H., Yu D., Zhao G., Lin L., Tang W., Yu H., Bao S., Xie Q. (2012). miR-15b and miR-16 regulate TNF mediated hepatocyte apoptosis via BCL2 in acute liver failure. Apoptosis.

[53] Yu D.S., An F.M., Gong B.D., Xiang X.G., Lin L.Y., Wang H., Xie Q. (2012). The regulatory role of microRNA-1187 in TNF-α-mediated hepatocyte apoptosis in acute liver failure. Int. J. Mol. Med..

[54] Sharma A.D., Narain N., Händel E.M., Iken M., Singhal N., Cathomen T., Manns M.P., Schöler H.R., Ott M., Cantz T. (2011). MicroRNA-221 regulates FAS-induced fulminant liver failure. Hepatology.

[55] Yuan Q., Loya K., Rani B., Möbus S., Balakrishnan A., Lamle J., Cathomen T., Vogel A., Manns M.P., Ott M. (2013). MicroRNA-221 overexpression accelerates hepatocyte proliferation during liver regeneration. Hepatology.

[56] Kalluri R., Weinberg R.A. (2009). The basics of epithelial-mesenchymal transition. J. Clin. Investig..

[57] Noetel A., Kwiecinski M., Elfimova N., Huang J., Odenthal M. (2012). microRNA are Central Players in Anti- and Profibrotic Gene Regulation during Liver Fibrosis. Front. Physiol..

[58] He Y., Huang C., Zhang S.P., Sun X., Long X.R., Li J. (2012). The potential of microRNAs in liver fibrosis. Cell Signal.

[59] Hu W., Coller J. (2012). What comes first: Translational repression or mRNA degradation? The deepening mystery of microRNA function. Cell Res..

[60] Djuranovic S., Nahvi A., Green R. (2012). miRNA-mediated gene silencing by translational repression followed by mRNA deadenylation and decay. Science.

[61] Bazzini A.A., Lee M.T., Giraldez A.J. (2012). Ribosome profiling shows that miR-430 reduces translation before causing mRNA decay in zebrafish. Science.

[62] Krek A., Grün D., Poy M.N., Wolf R., Rosenberg L., Epstein E.J., MacMenamin P., da Piedade I., Gunsalus K.C., Stoffel M. (2005). Combinatorial microRNA target predictions. Nat. Genet..

[63] Valinezhad Orang A., Safaralizadeh R., Kazemzadeh-Bavili M. (2014). Mechanisms of miRNA-Mediated Gene Regulation from Common Downregulation to mRNA-Specific Upregulation. Int. J. Genom..

[64] Gholipour N., Ohradanova-Repic A., Ahangari G. (2018). A novel report of MiR-4301 induces cell apoptosis by negatively regulating DRD2 expression in human breast cancer cells. J. Cell Biochem..

[65] Avval A.J., Majd A., Gholipour N., Noghabi K.A., Ohradanova-Repic A., Ahangari G. (2019). An Inventive Report of Inducing Apoptosis in Non-Small Cell Lung Cancer (NSCLC) Cell Lines by Transfection of MiR-4301. Anticancer Agents Med. Chem..

[66] Zhang H.M., Li H., Wang G.X., Wang J., Xiang Y., Huang Y., Shen C., Dai Z.T., Li J.P., Zhang T.C. (2020). MKL1/miR-5100/CAAP1 loop regulates autophagy and apoptosis in gastric cancer cells. Neoplasia.

[67] Yang L., Lin Z., Wang Y., Gao S., Li Q., Li C., Xu W., Chen J., Liu T., Song Z. (2018). MiR-5100 increases the cisplatin resistance of the lung cancer stem cells by inhibiting the Rab6. Mol. Carcinog..

[68] Lin H., Zhang R., Wu W., Lei L. (2021). miR-4454 Promotes Hepatic Carcinoma Progression by Targeting Vps4A and Rab27A. Oxid. Med. Cell Longev..

[69] Ye D., Zhang T., Lou G., Liu Y. (2018). Role of miR-223 in the pathophysiology of liver diseases. Exp. Mol. Med..

[70] Tian J., Li J., Bie B., Sun J., Mu Y., Shi M., Zhang S., Kong G., Li Z., Guo Y. (2021). MiR-3663-3p participates in the anti-hepatocellular carcinoma proliferation activity of baicalein by targeting SH3GL1 and negatively regulating EGFR/ERK/NF-κB signaling. Toxicol. Appl. Pharmacol..

[71] National Toxicology Program (1978). Bioassay of lasiocarpine for possible carcinogenicity. Natl. Cancer Inst. Carcinog. Tech. Rep. Ser..

[72] National Toxicology Program (2003). Toxicology and carcinogenesis studies of riddelliine (CAS No. 23246-96-0) in F344/N rats and B6C3F1 mice (gavage studies). Natl. Toxicol. Program Tech. Rep. Ser..

[73] Chen T., Mei N., Fu P.P. (2010). Genotoxicity of pyrrolizidine alkaloids. J. Appl. Toxicol..

[74] Jin X.H., Lu S., Wang A.F. (2020). Expression and clinical significance of miR-4516 and miR-21-5p in serum of patients with colorectal cancer. BMC Cancer.

[75] Cui T., Bell E.H., McElroy J., Becker A.P., Gulati P.M., Geurts M., Mladkova N., Gray A., Liu K., Yang L. (2019). miR-4516 predicts poor prognosis and functions as a novel oncogene via targeting PTPN14 in human glioblastoma. Oncogene.

[76] Chen S., Xu M., Zhao J., Shen J., Li J., Liu Y., Cao G., Ma J., He W., Chen X. (2020). MicroRNA-4516 suppresses pancreatic cancer development via negatively regulating orthodenticle homeobox 1. Int. J. Biol. Sci..

[77] Li O., Li Z., Tang Q., Li Y., Yuan S., Shen Y., Zhang Z., Li N., Chu K., Lei G. (2018). Long Stress Induced Non-Coding Transcripts 5 (LSINCT5) Promotes Hepatocellular Carcinoma Progression Through Interaction with High-Mobility Group AT-hook 2 and MiR-4516. Med. Sci. Monit..

[78] Ambros V., Bartel B., Bartel D.P., Burge C.B., Carrington J.C., Chen X., Dreyfuss G., Eddy S.R., Griffiths-Jones S., Marshall M. (2003). A uniform system for microRNA annotation. RNA.

[79] Van Keuren-Jensen K.R., Malenica I., Courtright A.L., Ghaffari L.T., Starr A.P., Metpally R.P., Beecroft T.A., Carlson E.W., Kiefer J.A., Pockros P.J. (2016). microRNA changes in liver tissue associated with fibrosis progression in patients with hepatitis C. Liver Int..

[80] Rane C.K., Minden A. (2019). P21 activated kinase signaling in cancer. Semin. Cancer Biol..

[81] Radu M., Semenova G., Kosoff R., Chernoff J. (2014). PAK signalling during the development and progression of cancer. Nat. Rev. Cancer.

[82] Advani S.J., Camargo M.F., Seguin L., Mielgo A., Anand S., Hicks A.M., Aguilera J., Franovic A., Weis S.M., Cheresh D.A. (2015). Kinase-independent role for CRAF-driving tumour radioresistance via CHK2. Nat. Commun..

[83] Arias-Romero L.E., Chernoff J. (2008). A tale of two Paks. Biol. Cell.

[84] Tu J., Zhao Z., Xu M., Chen M., Weng Q., Ji J. (2019). LINC00460 promotes hepatocellular carcinoma development through sponging miR-485-5p to up-regulate PAK1. Biomed. Pharmacother..

[85] Abdelfatah S., Naß J., Knorz C., Klauck S.M., Küpper J.H., Efferth T. (2021). Pyrrolizidine alkaloids cause cell cycle and DNA damage repair defects as analyzed by transcriptomics in cytochrome P450 3A4-overexpressing HepG2 clone 9 cells. Cell Biol. Toxicol..

