# Supplementary material for: Identification of microRNAs Implicated in Modulating Senecionine-Induced Liver Toxicity in HepaRG Cells
Source: Foods. 2022 Feb 12;11(4):532. doi: 10.3390/foods11040532 (PMC8871147; doi:10.3390/foods11040532)
Supplement: Supplementary file 1 [file foods-11-00532-s001.zip › foods-1581848-supplementary.pdf]

Supplementary Materials

Identification of microRNAs Implicated in Modulating Senecionine-Induced Liver Toxicity in HepaRG Cells

Anne-Margarethe Enge[1], Heike Sprenger[1], Albert Braeuning[1], Stefanie Hessel-Pras[1]

[1] Department of Food Safety, German Federal Institute for Risk Assessment, Max-Dohrn-Str. 8-10, 10589 Berlin, Germany

Corresponding Author:
Dr. Stefanie Hessel-Pras
German Federal Institute for Risk Assessment
Department of Food Safety
Max-Dohrn-Strasse 8-10
10589 Berlin
Germany
Phone: +49-30-18412-25203
E-mail: stefanie.hessel-pras@bfr.bund.de

Table S1: miRNAs investigated with miRCURY LNA miRNA custom PCR array, including 84 liver-specific miRNAs, 7 housekeeping genes, 1 interplate calibrator, 3 RNA isolation efficiency spike-in controls and 1 reverse transcription efficiency spike-in control.

no.	miRNA ID	remark
1	hsa-let-7a-5p	
2	hsa-let-7b-5p	
3	hsa-let-7c-5p	
4	hsa-let-7f-5p	
5	hsa-let-7g-5p	
6	hsa-miR-1-3p	
7	hsa-miR-100-5p	
8	hsa-miR-101-3p	
9	hsa-miR-106b-5p	
10	hsa-miR-107	
11	hsa-miR-122-5p	
12	hsa-miR-122-3p	
13	hsa-miR-125a-5p	
14	hsa-miR-125b-5p	
15	hsa-miR-126-5p	
16	hsa-miR-126-3p	
17	hsa-miR-1260a	
18	hsa-miR-142-5p	
19	hsa-miR-142-3p	
20	hsa-miR-143-3p	
21	hsa-miR-145-5p	
22	hsa-miR-145-3p	
23	hsa-miR-146a-5p	
24	hsa-miR-148a-3p	
25	hsa-miR-148b-3p	
26	hsa-miR-155-5p	
27	hsa-miR-15a-5p	
28	hsa-miR-16-5p	
29	hsa-miR-17-5p	
30	hsa-miR-192-5p	
31	hsa-miR-192-3p	

32	hsa-miR-194-5p	
33	hsa-miR-195-5p	
34	hsa-miR-199a-5p	
35	UniSp6	reverse transcription efficiency control
36	hsa-miR-199a-3p	
37	hsa-miR-19a-3p	
38	hsa-miR-19b-3p	
39	hsa-miR-200b-3p	
40	hsa-miR-20a-5p	
41	hsa-miR-21-5p	
42	hsa-miR-214-3p	
43	hsa-miR-22-3p	
44	hsa-miR-22-5p	
45	hsa-miR-221-3p	
46	hsa-miR-222-3p	
47	hsa-miR-223-3p	
48	hsa-miR-224-5p	
49	hsa-miR-23a-3p	
50	hsa-miR-23b-3p	
51	hsa-miR-24-3p	
52	hsa-miR-26a-5p	
53	hsa-miR-26b-5p	
54	hsa-miR-27a-3p	
55	hsa-miR-27b-3p	
56	hsa-miR-29a-3p	
57	hsa-miR-29a-5p	
58	hsa-miR-29b-3p	
59	hsa-miR-29c-3p	
60	hsa-miR-30a-5p	
61	hsa-miR-30b-5p	
62	hsa-miR-30c-5p	
63	hsa-miR-30d-5p	
64	hsa-miR-30e-5p	
65	hsa-miR-3183	

66	hsa-miR-34a-5p	
67	hsa-miR-34a-3p	
68	hsa-miR-3607-5p	
69	hsa-miR-3653-3p	
70	hsa-miR-3663-3p	
71	UniSp3	inter-plate calibration control
72	hsa-miR-375	
73	hsa-miR-3907	
74	hsa-miR-4286	
75	hsa-miR-4291	
76	hsa-miR-4301	
77	hsa-miR-4454	
78	hsa-miR-4516	
79	hsa-miR-451a	
80	hsa-miR-5100	
81	hsa-miR-5701	
82	hsa-miR-664b-3p	
83	hsa-miR-92a-3p	
84	hsa-miR-93-5p	
85	hsa-miR-940	
86	hsa-miR-99a-5p	
87	hsa-miR-103a-3p	housekeeping gene
88	hsa-miR-16-5p	housekeeping gene
89	hsa-miR-191-5p	housekeeping gene
90	hsa-miR-423-5p	housekeeping gene
91	hsa-miR-23a-3p	housekeeping gene
92	SNORD38B(hsa)	housekeeping gene
93	RNU1A1(mmu, hsa)	housekeeping gene
94	UniSp5	miRNA isolation efficiency control
95	UniSp4	miRNA isolation efficiency control
96	UniSp2	miRNA isolation efficiency control

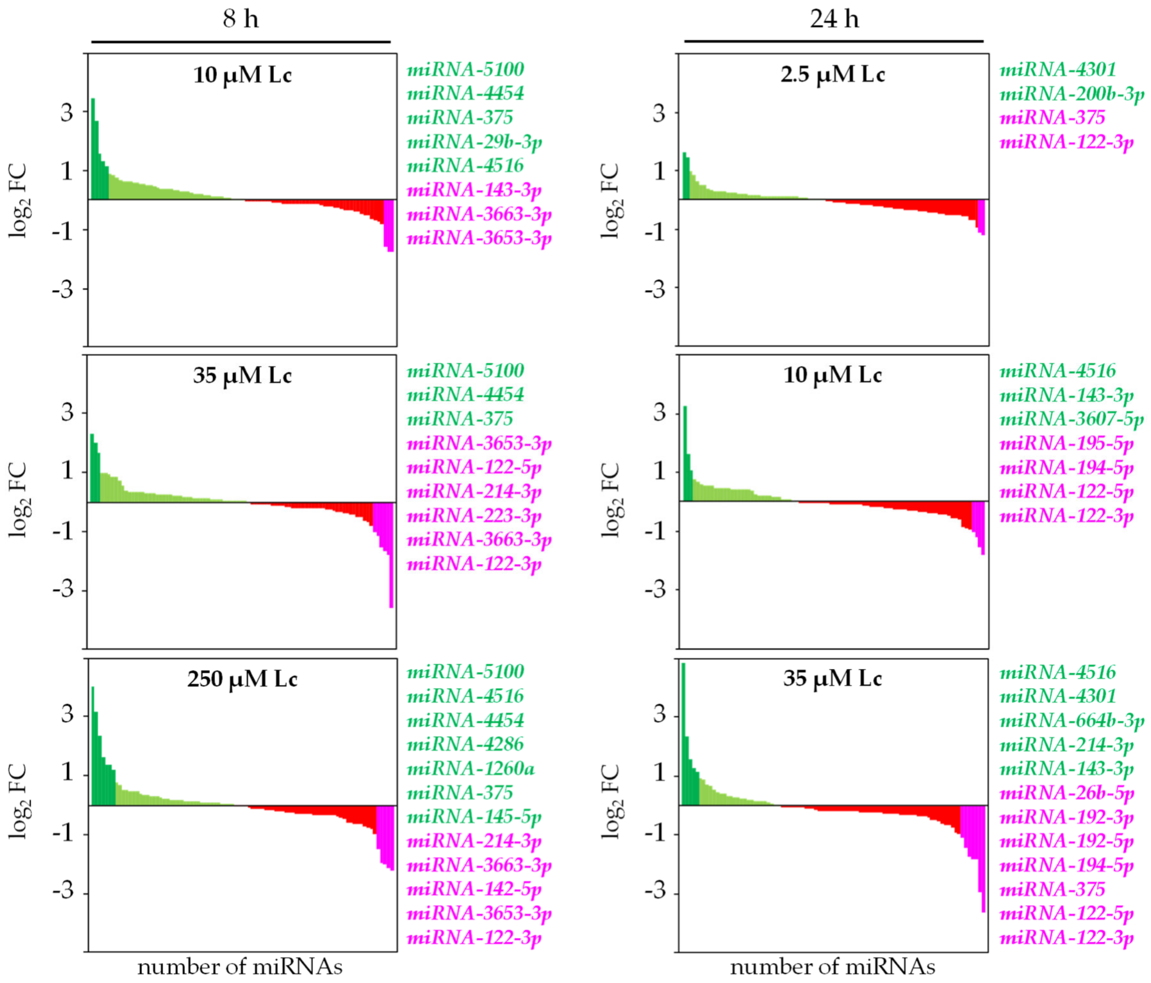

Figure S1: miRNA expression profile in differentiated HepaRG cells after exposure to Lc and determined with miRCURY LNA miRNA Array. HepaRG cells were incubated with 10 µM, 35 µM and 250 µM of Lc for 8 h or with 2.5 µM, 10 µM or 35 µM Lc for 24 h, respectively. The results were evaluated using the QIAGEN GeneGlobe miRCURY LNA miRNA PCR Data analysis software. This evaluation is in accordance with the $2^{-\Delta\Delta Ct}$ method and incorporates additional control such as internal plate calibrator, RNA isolation efficiency spike-in and reverse transcription efficiency spike-in controls. Compared to the control group, the number of deregulated miRNAs above 1 $\log_2$ FC is depicted in dark green and the number of miRNAs below 1 $\log_2$ FC is depicted in pink.

Table S2: miRNA expression in differentiated HepaRG cells after exposure to 35 μM Sc at 5 different time-points. The results were evaluated according to the $2^{-\Delta\Delta Ct}$ method. Results of three independent biological replicated are shown as mean and SD in log_2 FC. Statistical analysis was performed using Student's t-test as this is a case of single comparison analysis. That is, each miRNA expression value of one time point was compared to the solvent control (ctrl) of this respective time point. Subsequently, p-values were FDR-adjusted.

	ctrl		treatment		p-value
**gene names**	**mean**	**SD**	**mean**	**SD**	
miR-4434_2 h	0.00	0.00	0.06	0.29	7.6E-01
miR-4434_4 h	0.00	0.00	0.12	0.05	2.5E-02
miR-4434_6 h	0.00	0.00	0.45	0.10	3.9E-03
miR-4434_8 h	0.01	0.00	1.54	0.40	6.3E-03
miR-4434_24 h	0.01	0.01	3.06	0.90	2.9E-02
miR-4301_2 h	0.01	0.01	0.24	0.34	5.5E-01
miR-4301_4 h	0.02	0.01	1.09	0.58	4.4E-02
miR-4301_6 h	0.02	0.04	1.94	0.08	1.9E-05
miR-4301_8 h	0.00	0.00	3.00	1.12	1.7E-02
miR-4301_24 h	0.01	0.01	1.80	1.22	9.0E-02
miR-5100_2 h	0.00	0.00	0.33	0.30	5.5E-01
miR-5100_4 h	0.01	0.02	2.15	0.48	5.4E-03
miR-5100_6 h	0.03	0.06	2.58	0.73	6.8E-03
miR-5100_8 h	0.00	0.00	2.66	0.59	5.1E-03
miR-5100_24 h	0.00	0.00	1.40	1.04	9.3E-02
miR-4454_2 h	0.01	0.01	0.38	0.45	5.5E-01
miR-4454_4 h	0.01	0.02	1.23	0.22	4.8E-03
miR-4454_6 h	0.04	0.06	1.99	0.64	8.9E-03
miR-4454_8 h	0.00	0.00	2.50	0.50	5.1E-03
miR-4454_24 h	0.00	0.00	1.24	1.09	1.2E-01
miR-223_2 h	0.01	0.01	0.12	0.55	7.6E-01
miR-223_4 h	0.03	0.03	0.09	0.10	4.2E-01
miR-223_6 h	0.02	0.02	0.40	0.03	2.1E-04
miR-223_8 h	0.01	0.01	1.20	0.55	2.7E-02
miR-223_24 h	0.01	0.01	0.84	0.36	3.0E-02
miR-3663-3p_2 h	0.01	0.01	0.26	0.38	5.5E-01
miR-3663-3p_4 h	0.01	0.01	0.22	0.08	2.4E-02
miR-3663-3p_6 h	0.01	0.01	-0.10	0.17	4.0E-01

miR-3663-3p_8 h	0.00	0.00	0.62	0.30	2.9E-02
miR-3663-3p_24 h	0.01	0.01	0.75	0.29	3.0E-02
miR-122-3p_2 h	0.00	0.01	0.22	0.45	6.4E-01
miR-122-3p_4 h	0.02	0.01	0.02	0.09	9.7E-01
miR-122-3p_6 h	0.00	0.00	-0.07	0.35	7.5E-01
miR-122-3p_8 h	0.01	0.01	0.34	0.62	4.1E-01
miR-122-3p_24 h	0.01	0.01	-1.22	0.54	3.0E-02

Table S3: Target gene expression in differentiated HepaRG cells after exposure to 35 µM of Sc for 5 time points. The results were evaluated according to the $2^{-\Delta\Delta Ct}$ method. Results are shown as log_2 FC and as mean and SD of three individual replicates. Statistical analysis was performed using Student's t-test as this is a case of single comparison analysis. That is, each gene expression value of one time point was compared to the solvent control (ctrl) of this respective time point. Subsequently, data were FDR-adjusted.

	ctrl		treatment		p-value
**gene names**	**mean**	**SD**	**mean**	**SD**	
RACGAP1_2 h	0.00	0.00	-0.50	0.67	3.5E-01
RACGAP1_4 h	0.00	0.00	0.15	0.18	2.8E-01
RACGAP1_6 h	0.00	0.01	0.14	0.04	1.6E-02
RACGAP1_8 h	0.01	0.01	0.27	0.09	1.3E-02
RACGAP1_24 h	0.00	0.00	2.51	0.30	7.0E-04
FBXW7_2 h	0.10	0.16	0.20	0.57	8.1E-01
FBXW7_4 h	0.00	0.00	-0.21	0.09	3.8E-02
FBXW7_6 h	0.00	0.00	-0.05	0.09	4.2E-01
FBXW7_8 h	0.00	0.01	0.22	0.04	2.0E-03
FBXW7_24 h	0.54	0.92	1.95	1.14	2.0E-01
CASP2_2 h	0.00	0.00	-0.19	0.13	1.4E-01
CASP2_4 h	0.00	0.00	0.11	0.08	9.7E-02
CASP2_6 h	0.00	0.01	0.28	0.16	4.6E-02
CASP2_8 h	0.01	0.02	0.80	0.14	2.3E-03
CASP2_24 h	0.00	0.00	1.58	0.16	4.8E-04
SLC25A29_2 h	0.00	0.01	0.10	0.21	5.6E-01
SLC25A29_4 h	0.01	0.01	0.54	0.13	6.0E-03
SLC25A29_6 h	0.00	0.00	0.67	0.08	7.2E-04
SLC25A29_8 h	0.00	0.00	0.60	0.15	4.3E-03
SLC25A29_24 h	0.00	0.00	0.61	0.20	1.3E-02
REL_2 h	0.01	0.01	-0.51	0.06	2.0E-03
REL_4 h	0.00	0.00	0.15	0.19	2.8E-01
REL_6 h	0.00	0.00	0.29	0.16	4.6E-02
REL_8 h	0.01	0.01	0.45	0.15	1.2E-02
REL_24 h	0.01	0.01	0.26	0.06	5.4E-03
ATP7A_2 h	0.00	0.00	-0.17	0.23	1.4E-01
ATP7A_4 h	0.00	0.00	-0.20	0.20	4.6E-03
ATP7A_6 h	0.00	0.00	-0.16	0.09	2.4E-02

ATP7A_8 h	0.00	0.01	-0.43	0.23	2.1E-02
ATP7A_24 h	0.00	0.00	0.23	0.27	1.8E-01
PRKAR1A_2 h	0.01	0.01	-0.04	0.38	8.6E-01
PRKAR1A_4 h	0.00	0.00	0.45	0.13	1.2E-02
PRKAR1A_6 h	0.00	0.00	0.27	0.20	9.6E-02
PRKAR1A_8 h	0.01	0.01	0.67	0.11	2.0E-03
PRKAR1A_24 h	0.00	0.00	0.20	0.16	1.4E-01
SFXN1_2 h	0.01	0.00	-0.71	0.49	1.4E-01
SFXN1_4 h	0.00	0.00	-0.15	0.15	2.1E-01
SFXN1_6 h	0.00	0.01	-0.02	0.15	8.1E-01
SFXN1_8 h	0.01	0.01	0.44	0.13	7.1E-03
SFXN1_24 h	0.01	0.01	0.17	0.10	7.7E-02
BOK_2 h	0.00	0.00	-0.18	0.26	3.5E-01
BOK_4 h	0.00	0.00	-0.03	0.08	5.7E-01
BOK_6 h	0.00	0.00	0.36	0.06	2.1E-03
BOK_8 h	0.01	0.01	0.66	0.16	4.3E-03
BOK_24 h	0.00	0.00	0.12	0.07	6.2E-02
ATP6V1H_2 h	0.02	0.02	-0.80	0.52	1.4E-01
ATP6V1H_4 h	-0.08	0.15	-0.22	0.12	3.5E-01
ATP6V1H_6 h	0.01	0.01	-0.41	0.15	1.8E-02
ATP6V1H_8 h	0.03	0.02	0.08	0.22	7.2E-01
ATP6V1H_24 h	0.00	0.00	0.05	0.17	6.6E-01
LEPROT_2 h	0.00	0.00	-0.15	0.11	1.4E-01
LEPROT_4 h	0.01	0.01	0.22	0.05	6.0E-03
LEPROT_6 h	0.00	0.00	0.20	0.13	6.6E-02
LEPROT_8 h	0.01	0.01	0.41	0.08	2.6E-03
LEPROT_24 h	0.00	0.00	-0.11	0.08	1.1E-01
IFIH1_2 h	0.00	0.00	-0.09	0.23	5.6E-01
IFIH1_4 h	0.02	0.04	-0.26	0.18	9.7E-02
IFIH1_6 h	0.01	0.01	-0.49	0.26	4.6E-02
IFIH1_8 h	0.01	0.01	-0.72	0.16	3.2E-03
IFIH1_24 h	0.00	0.00	-0.11	0.16	3.0E-01
ACVR2A_2 h	0.00	0.00	-0.17	0.13	3.5E-01
ACVR2A_4 h	0.00	0.00	0.29	0.06	2.1E-01

ACVR2A_6 h	0.01	0.01	0.53	0.21	4.6E-02
ACVR2A_8 h	0.00	0.00	0.45	0.20	3.7E-02
ACVR2A_24 h	0.00	0.00	-0.12	0.12	2.4E-01
STX17_2 h	0.01	0.01	-0.47	0.24	8.0E-02
STX17_4 h	0.00	0.00	-0.29	0.14	4.6E-02
STX17_6 h	0.00	0.00	-0.22	0.15	7.3E-02
STX17_8 h	0.00	0.00	-0.12	0.13	1.8E-01
STX17_24 h	0.00	0.00	-0.27	0.04	1.4E-03
PATZ1_2 h	0.00	0.00	-0.25	0.09	2.7E-02
PATZ1_4 h	0.01	0.01	-0.28	0.29	2.2E-01
PATZ1_6 h	0.00	0.01	-0.39	0.13	1.6E-02
PATZ1_8 h	0.00	0.00	-0.41	0.23	4.4E-02
PATZ1_24 h	0.00	0.00	-0.36	0.16	2.9E-02
PAK1_2 h	0.00	0.01	-0.17	0.16	2.2E-01
PAK1_4 h	0.00	0.00	-0.57	0.22	2.6E-02
PAK1_6 h	0.00	0.00	-0.51	0.21	2.5E-02
PAK1_8 h	0.01	0.01	-0.51	0.20	1.6E-02
PAK1_24 h	0.00	0.00	-0.49	0.13	6.5E-03
FOXO1_2 h	0.00	0.01	-0.21	0.30	3.5E-01
FOXO1_4 h	0.00	0.00	-0.51	0.10	4.5E-03
FOXO1_6 h	0.00	0.00	-0.48	0.10	2.7E-03
FOXO1_8 h	0.00	0.00	-0.53	0.13	3.9E-03
FOXO1_24 h	0.00	0.01	-0.68	0.25	1.7E-02
SLC4A4_2 h	0.00	0.00	-0.42	0.20	7.1E-02
SLC4A4_4 h	0.00	0.00	-0.55	0.11	4.5E-03
SLC4A4_6 h	0.00	0.01	-0.78	0.15	2.7E-03
SLC4A4_8 h	0.00	0.00	-0.98	0.18	2.3E-03
SLC4A4_24 h	0.00	0.00	-0.70	0.10	9.5E-04
AOX1_2 h	0.00	0.00	-0.54	0.11	1.2E-02
AOX1_4 h	0.00	0.01	-0.74	0.07	6.3E-04
AOX1_6 h	0.00	0.00	-1.10	0.21	2.7E-03
AOX1_8 h	0.00	0.00	-1.31	0.13	1.5E-03
AOX1_24 h	0.00	0.00	-1.87	0.31	1.4E-03
GAS2_2 h	0.00	0.00	-0.10	0.04	5.7E-02

GAS2_4 h	0.00	0.00	0.09	0.19	4.7E-01
GAS2_6 h	0.01	0.01	-0.75	0.04	1.2E-04
GAS2_8 h	0.01	0.00	-1.58	0.23	1.9E-03
GAS2_24 h	0.00	0.00	-2.34	0.24	4.8E-04
ST6GAL1_2 h	0.00	0.00	-0.76	0.20	1.8E-02
ST6GAL1_4 h	0.00	0.01	-1.13	0.11	6.3E-04
ST6GAL1_6 h	0.00	0.00	-1.87	0.15	3.2E-04
ST6GAL1_8 h	0.01	0.01	-2.68	0.36	1.9E-03
ST6GAL1_24 h	0.00	0.00	-4.84	0.11	3.3E-06

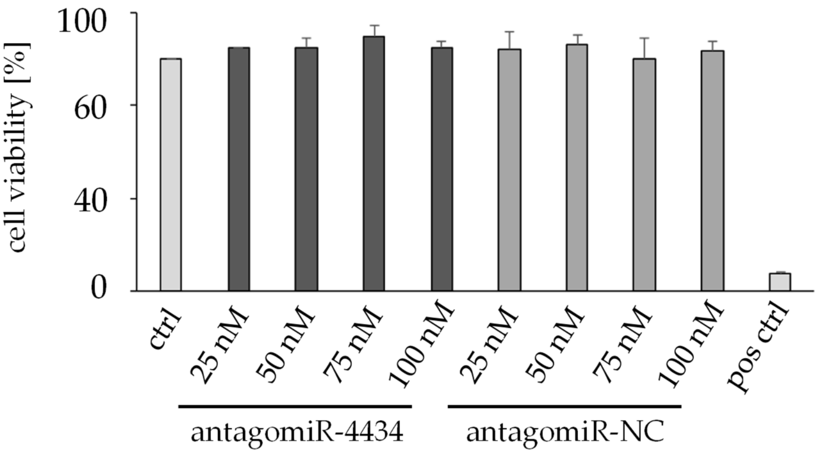

Figure S2: Cytotoxicity of differentiated HepaRG cells after antagomiR/antagomiR-NC- and Sc-incubation. Fully differentiated HepaRG cells were incubated with 35 µM of Sc and subsequently transiently transfected with 25 nM, 50 nM, 75 nM and 100 nM of antagomiR-4434 or antagomiR-NC, respectively. Cell viability was assessed using the NRU assay and is displayed as percent of solvent control (ctrl; 0.35% ACN and transfection reagent with water) as mean + SD of three independent experiments with five technical replicates each. 0.01% Triton X-100 was used as a positive control (pos ctrl).

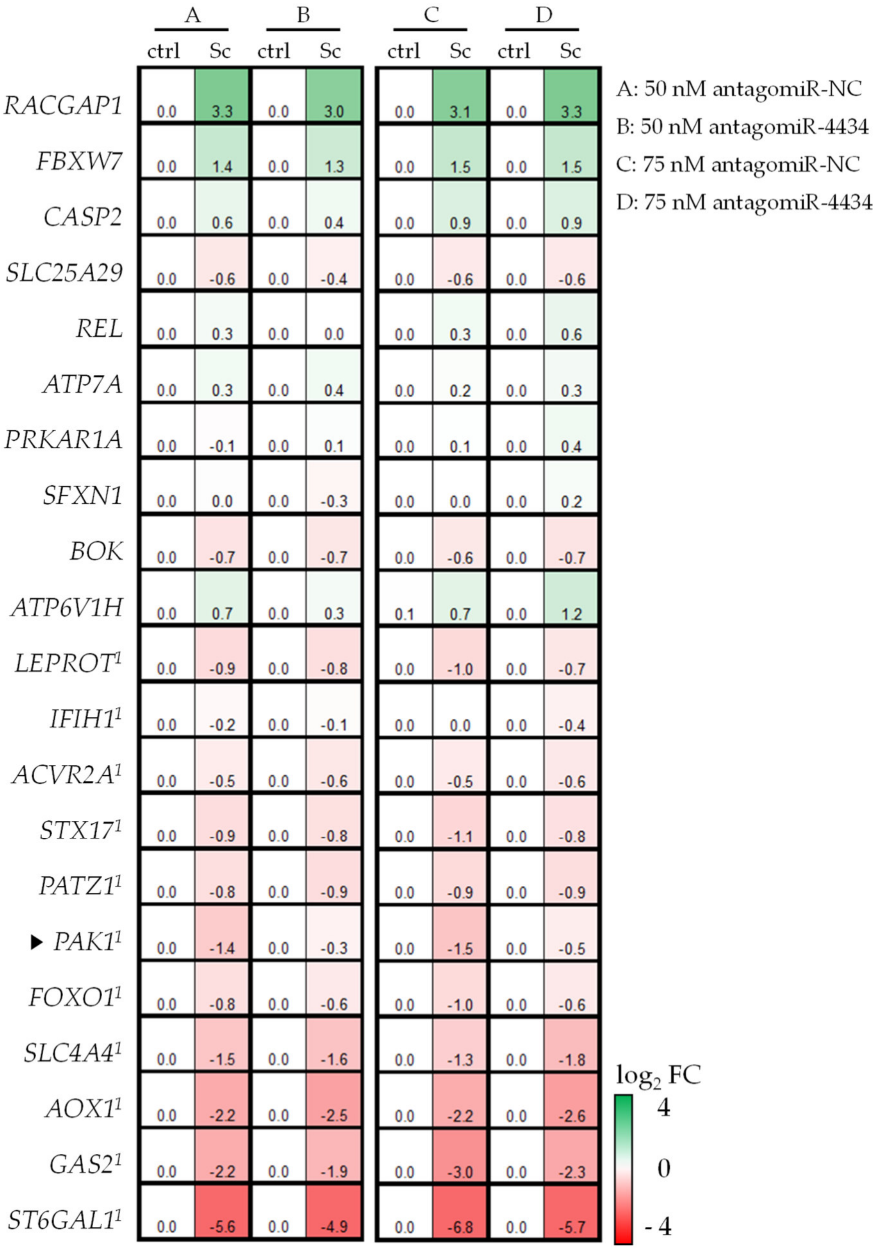

Figure S3: Target gene expression in differentiated HepaRG cells after exposure to 35 µM Sc and antagomiR-mediated inhibition of miR-4434 after 48 h. The results were evaluated according to the $2^{-\Delta\Delta Ct}$ method. After housekeeper-normalization, the values of the Sc-treated cells were referred to the respective solvent control (ctrl; 0.35% ACN). Noteworthy, the different treatments (antagomiR-4434 and antagomiR-NC) were referred to their respective solvent controls. AntagomiR/antagomiR-NC treatment was applied in the two concentrations 50 nM and 75 nM. Results are shown as $\log_2$ FC and as mean of three replicates. Upregulation or downregulation of gene expression is indicated in green or red, respectively, with increasing intensity of coloration. Mean values and standard deviations are summarized in the Supplementary Materials Table S4. Superscript number 1 indicates the target genes that show an opposite regulation to their annotated miRNAs. A and B: transfection with 50 nM antagomiR-NC or antagmiR-4434, C and D: transfection with 75 nM antagomiR-NC or antagmiR-4434. Black arrow indicates target gene whose downregulation was significantly attenuated upon miR-4434 inhibition (see Figure 5).

Table S4: Target gene expression in differentiated HepaRG cells after exposure to 35 µM Sc and antagomiR-mediated inhibition of miR-4434 after 48 h. The results were evaluated according to the $2^{-\Delta\Delta Ct}$ method and as described above. Results are shown as $\log_2$ FC and as mean and SD of three individual replicates. **(a)** A: Solvent control and Sc-treatment (35 µM, 48 h) and transfection with 50 nM antagomiR-NC. B: Solvent control and Sc-treatment (35 µM, 48 h) and transfection with 50 nM antagomiR-4434. **(b)** C: Solvent control and Sc-treatment (35 µM, 48 h) and transfection with 75 nM antagomiR-NC. D: Solvent control and Sc-treatment (35 µM, 48 h) and transfection with 75 nM antagomiR-4434.

**(a)**	(A) solvent control and antagomiR-NC (50 nM)		(A) Sc-treatment and antagomiR-NC (50 nM)		(B) solvent control and antagomiR-4434 (50 nM)		(B) Sc-treatment and antagomiR-4434 (50 nM)	
**gene names**	**mean**	**SD**	**mean**	**SD**	**mean**	**SD**	**mean**	**SD**
RACGAP1	0.01	0.00	3.32	0.37	0.01	0.01	3.00	0.16
FBXW7	0.00	0.00	1.44	0.39	0.00	0.00	1.32	0.18
CASP2	0.00	0.00	0.58	0.28	0.01	0.01	0.37	0.49
SLC25A29	0.00	0.00	-0.60	0.18	0.01	0.01	-0.40	0.09
REL	0.01	0.01	0.29	0.33	0.01	0.01	-0.01	0.18
ATP7A	0.02	0.02	0.34	0.51	0.01	0.01	0.35	0.07
PRKAR1A	0.02	0.02	-0.08	0.57	0.02	0.03	0.13	0.35
SFXN1	0.04	0.06	-0.04	0.53	0.00	0.01	-0.27	0.07
BOK	0.00	0.00	-0.73	0.14	0.00	0.00	-0.65	0.07
ATP6V1H	0.02	0.03	0.74	0.71	0.04	0.08	0.29	0.37
LEPROT	0.00	0.00	-0.94	0.55	0.00	0.00	-0.84	0.35
IFIH1	0.00	0.00	-0.17	0.27	0.02	0.01	-0.09	0.19
ACVR2A	0.01	0.01	-0.49	0.10	0.01	0.02	-0.63	0.30
STX17	0.00	0.00	-0.89	0.32	0.01	0.01	-0.82	0.19
PATZ1	0.00	0.00	-0.84	0.26	0.00	0.00	-0.88	0.17
PAK1	0.00	0.00	-1.38	0.26	0.00	0.00	-0.34	0.79
FOXO1	0.00	0.00	-0.84	0.33	0.01	0.01	-0.57	0.05
SLC4A4	0.00	0.01	-1.54	0.10	0.00	0.01	-1.58	0.54
AOX1	0.00	0.00	-2.24	0.29	0.00	0.00	-2.47	0.47
GAS2	0.00	0.00	-2.21	0.36	0.01	0.01	-1.94	0.61
ST6GAL1	0.00	0.00	-5.55	0.92	0.00	0.00	-4.86	1.04

**(b)**	(C) solvent control and antagomiR-NC (75 nM)		(C) Sc-treatment and antagomiR-NC (75 nM)		(D) solvent control and antagomiR-4434 (75 nM)		(D) Sc-treatment and antagomiR-4434 (75 nM)	
**gene names**	**mean**	**SD**	**mean**	**SD**	**mean**	**SD**	**mean**	**SD**
RACGAP1	0.00	0.00	3.09	0.44	0.00	0.00	3.32	0.16
FBXW7	0.00	0.00	1.49	0.39	0.00	0.00	1.51	0.14
CASP2	0.04	0.06	0.95	0.16	0.00	0.00	0.92	0.08
SLC25A29	0.01	0.00	-0.57	0.09	0.00	0.00	-0.56	0.16
REL	0.00	0.00	0.33	0.37	0.01	0.02	0.56	0.16
ATP7A	0.01	0.01	0.15	0.29	0.00	0.00	0.32	0.21
PRKAR1A	0.02	0.02	0.05	0.30	0.01	0.01	0.40	0.31
SFXN1	0.05	0.04	0.00	0.04	0.00	0.00	0.23	0.16
BOK	0.00	0.00	-0.61	0.19	0.00	0.01	-0.71	0.07
ATP6V1H	0.07	0.08	0.74	0.42	0.03	0.02	1.21	0.34
LEPROT	0.00	0.00	-0.98	0.59	0.01	0.01	-0.66	0.22
IFIH1	0.00	0.00	0.05	1.03	0.00	0.00	-0.37	0.39
ACVR2A	0.01	0.00	-0.55	0.14	0.00	0.00	-0.59	0.15
STX17	0.01	0.01	-1.05	0.05	0.00	0.00	-0.78	0.26
PATZ1	0.00	0.00	-0.95	0.33	0.00	0.00	-0.86	0.12
PAK1	0.00	0.00	-1.54	0.08	0.00	0.00	-0.48	0.08
FOXO1	0.00	0.00	-0.96	0.32	0.00	0.00	-0.58	0.07
SLC4A4	0.01	0.01	-1.29	0.18	0.00	0.00	-1.76	0.35
AOX1	0.00	0.00	-2.21	0.13	0.00	0.00	-2.58	0.24
GAS2	0.01	0.01	-2.95	0.15	0.01	0.01	-2.30	0.41
ST6GAL1	0.00	0.00	-6.83	0.40	0.00	0.00	-5.72	0.98
